# Covalent Inhibitors of S100A4 Block the Formation of a Pro-Metastasis Non-Muscle Myosin 2A Complex

**DOI:** 10.1021/acs.jmedchem.4c01320

**Published:** 2024-10-19

**Authors:** Charline Giroud, Tamas Szommer, Carmen Coxon, Octovia Monteiro, Thomas Grimes, Tryfon Zarganes-Tzitzikas, Thomas Christott, James Bennett, Karly Buchan, Paul E. Brennan, Oleg Fedorov

**Affiliations:** † Centre for Medicines Discovery, Nuffield Department of Medicine, NDM Research building, Old Road Campus, Oxford OX3 7FZ, U.K.; ‡ Alzheimer’s Research UK Oxford Drug Discovery Institute, NDM Research Building, Old Road Campus, Oxford OX3 7FZ, U.K.

## Abstract

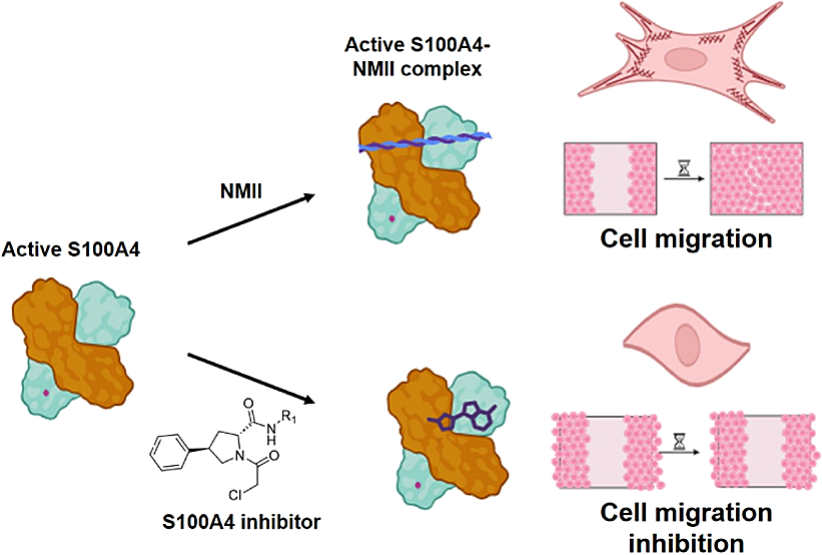

The S100 protein family functions as protein–protein interaction adaptors regulated by Ca^2+^ binding. Formation of various S100 complexes plays a central role in cell functions, from calcium homeostasis to cell signaling, and is implicated in cell growth, migration, and tumorigenesis. We established a suite of biochemical and cellular assays for small molecule screening based on known S100 protein–protein interactions. From 25 human S100 proteins, we focused our attention on S100A4 because of its well-established role in cancer progression and metastasizes by interacting with nonmuscle myosin II (NMII). We identified several potent and selective inhibitors of this interaction and established the covalent nature of binding, confirmed by mass spectrometry and crystal structures. **5b** showed on-target activity in cells and inhibition of cancer cell migration. The identified S100A4 inhibitors can serve as a basis for the discovery of new cancer drugs operating via a novel mode of action.

## Introduction

Metastasis is directly responsible for an overwhelming majority of cancer deaths, with the exact percentage depending on the cancer type.^1^ Understanding the molecular mechanisms and potential targets for pharmacological intervention is therefore vitally important for developing new cancer therapies.^2,3^

The essential step in the metastatic process is the epithelial–mesenchymal transition (EMT).^4^ Among many important molecular players in this process is S100A4. When first discovered in 1993, it was tellingly named metastasin l (Mts1), a protein with an important role in the control of metastasis in mouse tumors.^5^ S100A4 is a member of S100 proteins, which have been considered as a marker of cancer and a poor prognostic of survival for a long time; however, increasing studies have shown that they play a major role in tumorigenesis and that they would be ideal new therapeutic targets.^6,7^ Numerous in vivo experiments have shown that S100A4-overexpression in cell-transplants increased their cell growth as well as their metastatic phenotype, whereas reduction or knock out of S100A4 expression was associated with a metastatic profile loss.^8−15^ Overexpression of S100A4 in benign tumor cells induces a metastatic phenotype in nude mice, whereas S100A4 silencing using antisense RNA reduced their metastatic potential.^9,10^ In rats, injection of a non-metastasizing tumor cell-line overexpressing an S100A4 analogue produced tumors with a shorter latent period and the ability to metastasize compared to the control cancer cells.^8^ These studies strongly suggest that S100A4 has the ability to turn on and off cancer growth and the metastatic profile.

The S100 calcium-binding protein family consists of low molecular weight proteins (∼11 kDa) expressed by vertebrates in specific cell types and tissues. S100 proteins generally form homodimers in which each subunit is made of four helices arranged in two calcium-binding loops, also known as EF-hand domains. Upon calcium flux into the cell, calcium bound by EF-hands triggers conformational changes in the dimer that expose hydrophobic regions and result in its ability to interact with protein targets. This mechanism refers to the calcium-dependent activation or calcium switch. S100 proteins bind a diverse range of protein targets and regulate a large number of protein functions. A lot of effort has been made to find a protein epitope, or motif, recognized by S100 proteins that would explain their specificity of interaction with their target. A screening of a bacteriophage random-peptide display-library allowed Ivanenkov et al. to find the first consensus sequence for S100B proteins.^16^ This sequence, also known as TRTK-12, has been shown later to be recognized by a wider set of S100 proteins and has long been considered as a universal S100 protein partner.^17,18^

The mechanism by which S100A4 exerts its metastatic action is not yet clearly understood, but NMIIA (the nonmuscle myosin 2A) has been suggested as a potential target involved in this process.^19−21^ The structure of the calcium-bound S100A4-NMII complex has shown an asymmetric interaction mode between S100A4 and NMII^22^, in which the α helix of NMII stretches along the S100A4 dimer, involving the calcium-dependent binding domain of each monomer. Other structural studies strongly suggest that the NMII coil–coiled structure untwists upon S100A4 interaction, leading to filament disassembly and disruption,^23^ promoting cell remodeling and motility.^24^

To date, only a few inhibitors are available for S100A4. Niclosamide inhibits S100A4 at its transcription level,^25,26^ and phenothiazine inhibits the S100A4 protein by inducing its oligomerization with an IC_50_ of around 100 μM.^27^ Pentamidine, an inhibitor of the S100-p53 interaction, has been shown to target S100A4 in vitro by interacting with the fourth helix in micromolar-range concentrations and shows antiproliferative activities in cancer cells.^28^ Interestingly, a monoclonal S100A4 antibody has been reported to have activity in a skin fibrosis model.^29^ Most recently, new S100A4 inhibitors were published. Even though they have very weak affinity, approximately 50 μM, conversion to PROTAC enabled the suppression of S100A4 expression in the nanomolar range.^30^ This shows that developing potent small molecular weight S100A4 inhibitors is a feasible task.

In this study, we developed a protein–peptide interaction assay applicable for high-throughput screening for new S100 inhibitor discovery. We screened an in-house library of approximately 5 000 compounds and discovered phenylproline derivatives which inhibit S100A4-NMII interactions in vitro in the submicromolar range. We characterized their mechanism of inhibition and crystal structures and found that they covalently and specifically react with cysteine 81 of S100A4, a residue that sits in the NMII binding site. The activity of phenylproline derivatives also inhibits S100A4-NMII interactions in vivo and shows a reduction of cell migration in treated cancer cells.

## Results

### AlphaScreen Assay for S100A Proteins and Pilot Library Screening

As a starting point to discover new S100 inhibitors, we used a peptide-displacement assay based on the Amplified Luminescent Proximity Homogeneous Assay (AlphaScreen) of a His-tagged protein interacting with a biotinylated peptide and its ability to be disrupted by small molecule inhibitors of protein interactions.^31^ We took advantage of the ability of several S100 proteins to interact with the TRTK-12 peptide.^32^ The optimal experimental conditions were determined by performing a protein versus peptide titration for each protein. Serial dilutions of purified 6His-S100 protein (S100A2, S100A4, S100A5, or S100B) were mixed with a serial dilution of a biotinylated TRTK-12 peptide in the presence of 1 mM calcium to allow conformational changes and activation of S100 proteins. The protein–peptide ratio that allows the highest signal to background, without signal saturation, was chosen as the assay condition for each protein to perform the first screening campaign (Figure S1 and Table S1). An internal diversity library of 4 989 small molecules was screened at a single-dose of 50 μM in duplicate in a 384-well plate format. The screening statistics are shown in Table S2. The four S100 protein screening experiments were performed in parallel. An inhibition of 80% of the complex formation was applied as a cutoff for the selection of the best hits as a first criterion. We also selected the hits based on their ability to show specificity for one protein; compounds showing 50% or more inhibition for other S100 proteins were not selected (Supporting Information).

**Figure 1:**
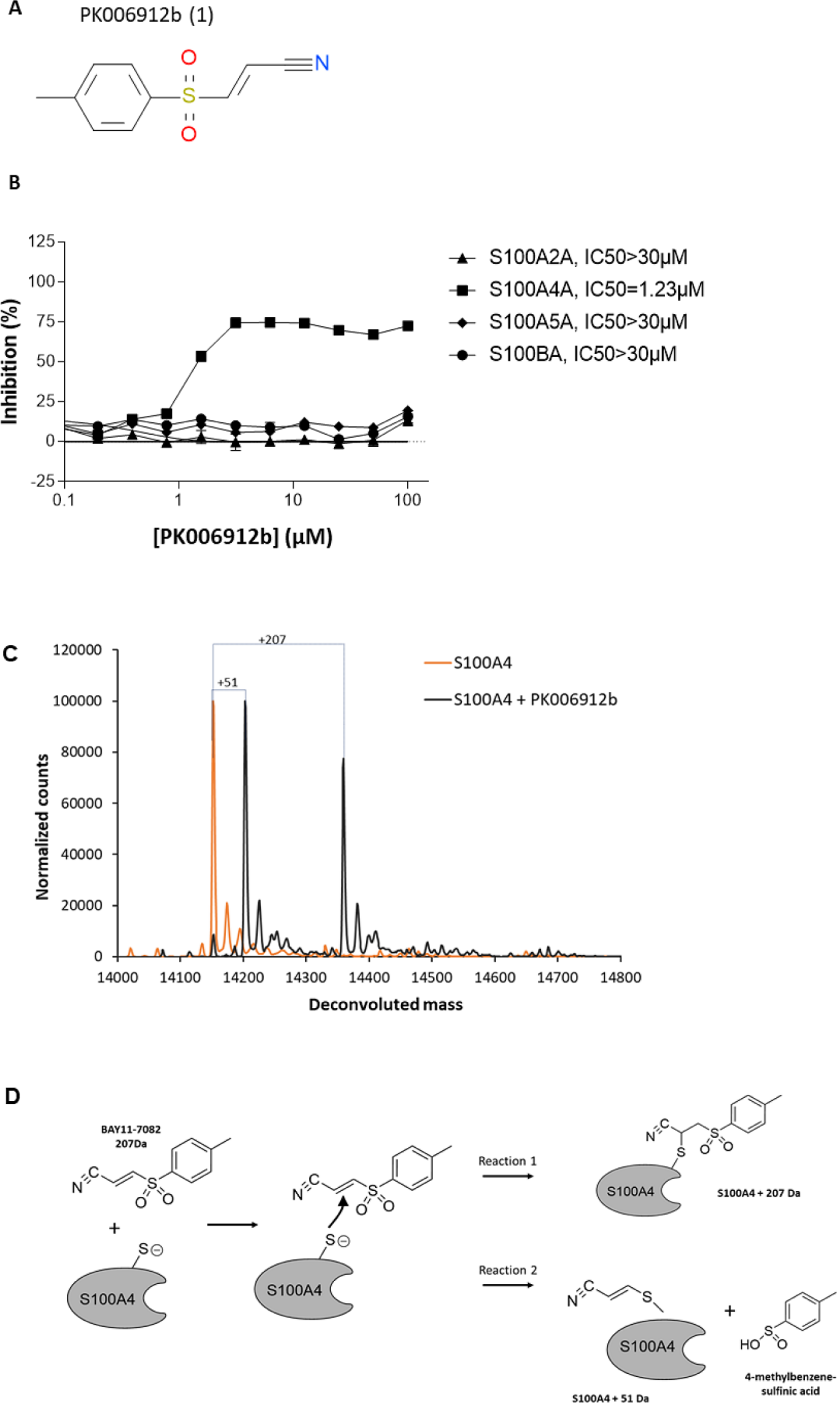
(A) PK006912b (aka **1** or BAY11–7082) has been (B) tested for IC_50_ determination against S100A2/A4/A5/B – TRTK peptide displacement. Compounds were tested in duplicate at a 50 μM top FAC. (C) Mass-spectrometry analysis of normalized counts vs mass of S100A4 treated with PK006912a at 100 μM (**1**), gray, compared to untreated S100A4 protein, orange. (D) Proposed reactions for S100A4 labeling by BAY11–7082 leading to 51 and 207 Da shift, via C3 and C2 positions, respectively.

The 15 best compounds of the library were moved forward for IC_50_ determination at a top assay concentration of 50 μM in duplicate. Almost all tested compounds showed IC_50_ higher than 5 μM, and only one compound, PK006912b (**1**) (Figure 1A), showed very high potency with an IC_50_ below 1 μM (Figure 1B). This compound, also known as BAY11–7082, or (*E*)-3-(4-methylphenylsulfonyl)-2-propenenitrile, is part of a compound family that covalently reacts with cysteines by conjugate addition.^33^ To confirm its mechanism of inhibition, we performed a protein labeling assay. S100A4 protein was incubated in the presence of BAY11–7082 and analyzed by mass spectrometry (Figure 1C). The mass spectrum of S100A4 shows two shifts of the protein mass, one of 51 Da and another of 207 Da, which corresponds to the molecular weight of BAY11–7082. Both types of adducts have been reported before with other proteins^34,35^ and are formed by two different reaction mechanisms (Figure 1D). Interestingly, only a single type of adduct, or *m*/*z* shift, was detected in each case, depending on the protein. As described by Strickson et al., in one case, the cysteine residue of the protein has reacted with BAY11–7082 by forming a covalent bond on the C3 carbon, resulting in a 51 Da shift of the protein mass and the elimination of 4-methylbenzene-sulfinic acid. On the other hand, the cysteine residue has reacted on the C2 carbon, forming a stable covalent adduct with a 207 Da *m*/*z* shift. S100A4 is the first example to our knowledge where both reactions can take place with a similar yield. It might indicate a relatively permissive environment of the reactive cysteine, which can be targeted by various cysteine-modifying warheads.^36^ Inspired by the discovery of BAY11–7082 as a potent covalent inhibitor, we focused our following work on discovering additional covalent inhibitors that would potentially provide improved potency and specificity.

### Covalent Library Screening

**Figure 2:**
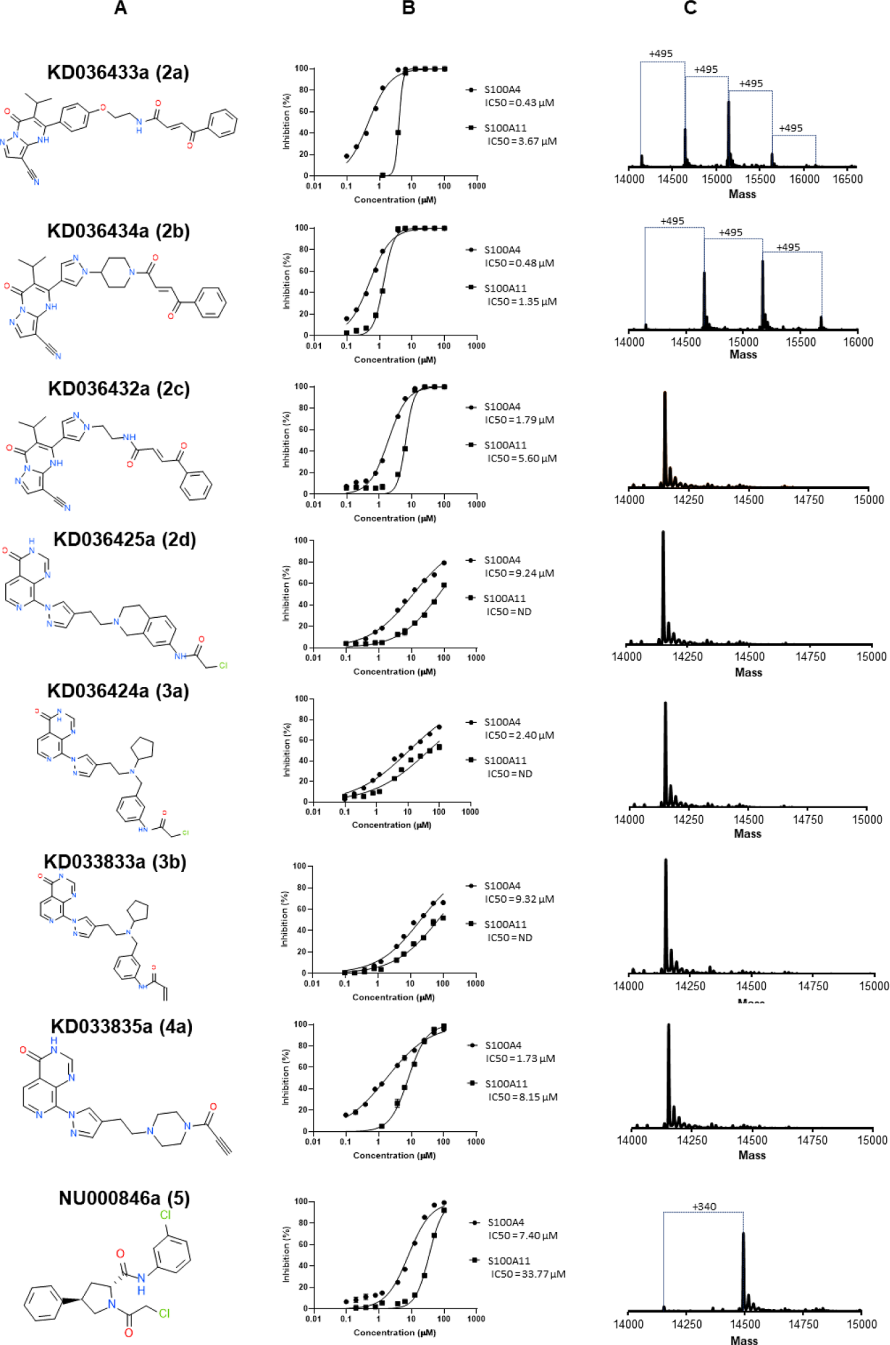
(A) Potential covalent inhibitors have been tested for (B) IC_50_ determination against S100A4-NMII and S100A11-AnII in the HTRF assay. (C) Their covalent activity was tested in parallel by mass spectrometry by incubating 10 μM of S100A4 protein with 100 μM of the inhibitor.

BAY11–7082 is a potent submicromolar inhibitor for S100A4, which belongs to a family of compounds that has been described to inhibit numerous proteins.^34,37−39^ This makes it a nonselective hit, which may be difficult to optimize for further applications. For future work, we decided to change the format from AlphaScreen to TR-FRET, which is known to be less prone to interference and generally more robust. Using the TR-FRET method, we tested the S100A4 interaction with a more specific and biologically relevant peptide, MPN, which mimics the MNII coil–coiled domain that is known to interact with S100A4.^23^ To control inhibitor specificity, we also performed in parallel a TR-FRET assay using S100A11 and an AnII-mimicking peptide.^40^ The choice of S100A11 was dictated by the presence of cysteine on the peptide binding site similar to S100A4 as determined by sequence and structure allignemnt of the proteins. Both assays were validated by dose–response experiments as described previously (suppl 3 and 4) and used for screening one of our internal libraries that contain potential covalent inhibitors developed as inhibitors of histone demethylase KDM5A^41^ or Nudix7 hydrolyze NUDT7.^42^ We took advantage of the small size of this library to directly perform the IC_50_ determination. Compounds were tested in duplicate in two independent experiments at 100 μM top concentration in the presence of 1 mM calcium. Nine compounds inhibited the S100A4-NMII interaction with an IC_50_ lower than 10 μM, and six compounds showed moderate specificity for S100A4. The compounds and their activity on S100A4-NMII and S100A11-AnII are shown in Figure 2A,B. To investigate their covalent activity, we then performed a protein labeling assay by incubating S100A4 in the presence of the inhibitors in parallel. Interestingly, five compounds did not show any covalent modification under our tested conditions, whereas KD036433a (**2a**) and KD036434a (**2b**), two phenylacryl compounds, showed multiple adduct additions characterized by multiple labeled peaks of the protein on the spectra (Figure 2C). NU000846a (**5**), a phenylproline derivative, showed S100A4 modification characterized by one shift of 340 Da. This modification strongly suggests a covalent reaction between S100A4 and the inhibitor combined with a loss of a chlorine. Based on this single shift seen on the spectrum, the NU000846a (**5**) compound was selected as a hit for further investigations.

### Validation of Additional Phenylproline Inhibitors

**Figure 3:**
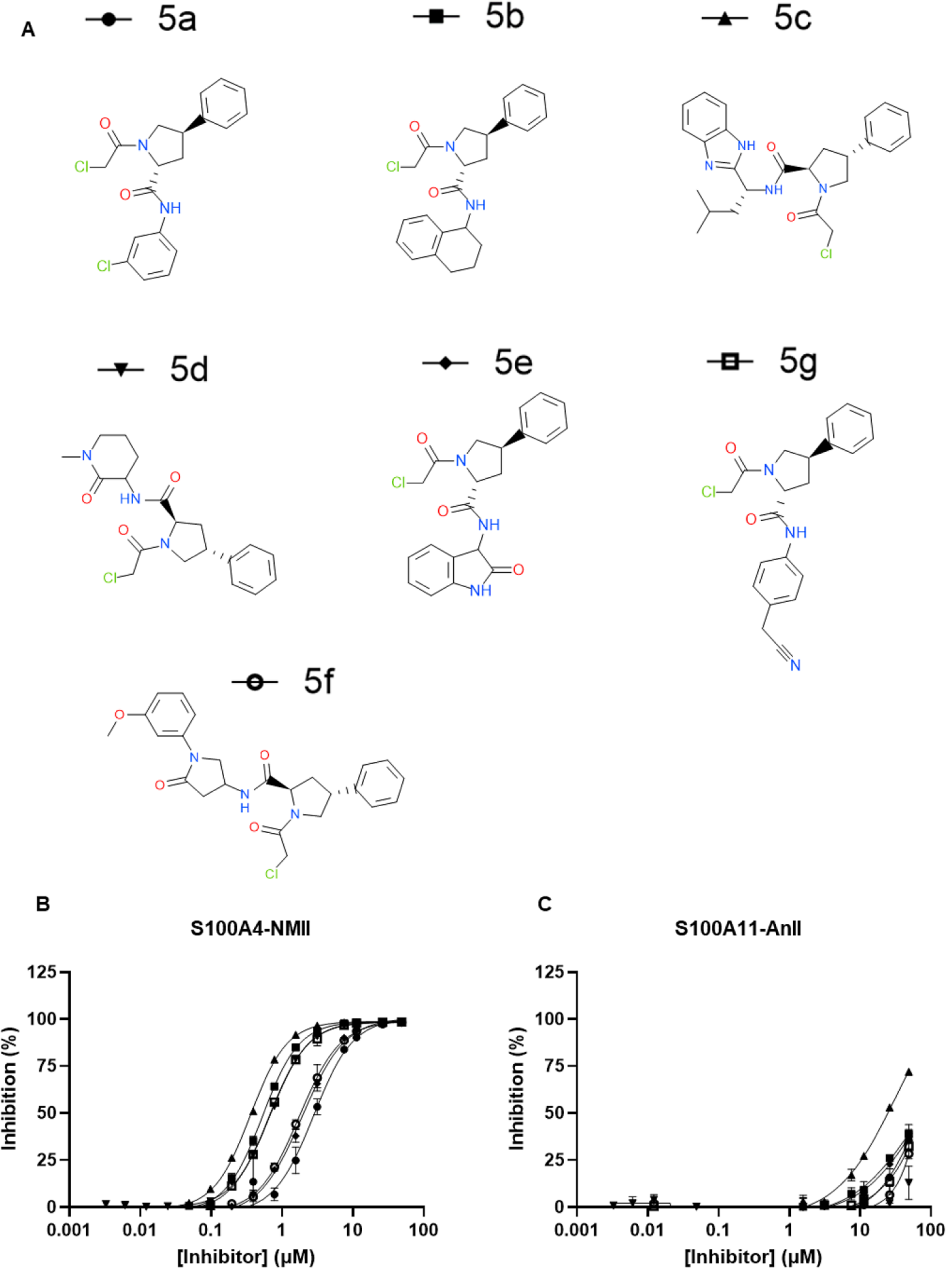
(A) Structure of phenylproline derivatives for S100A4 inhibitor optimization. (B) The compounds were tested in the HTRF assay against S100A4-NMII and (C) S100A11-AnII in duplicate at a top FAC of 48.75 μM in two independent experiments.

NU000846a (**5**) was the only compound in the preliminary selected covalent inhibitor library that showed selectivity for S100A4 over S100A11 (Table S3) and labeling a single cysteine. In order to make a potent S100A4 inhibitor, and based on its inhibition profile, further NU000486a derivatives were obtained from our in-house screening library (Figure 3A). A new stock solution of NU000846a was also acquired and named **5a**, and PK006912b (**1**) was added to the assay for comparison. The freshly prepared **5a** showed a slightly increased activity against S100A4-NMII compared to the previous batch of NU000846a (**5**), with a small reduction of the IC_50_ to 2.9 μM (Figure 3B). Six phenylproline derivatives inhibit the S100A4-NMII complex formation, with IC_50_ values below 1 μM, 0.38 μM for **5c**, and 0.48 μM for **5b** being the most active. All the new compounds tested in this experiment showed no detectable activity against S100A11-AnII, confirming their specificity for S100A4 (Figure 3C). Based on their activity improvement compared to the original inhibitor, **5a**, **5c**, and **5b** were selected for further characterizations.

### Characterization of Phenylproline Inhibitors

**Figure 4:**
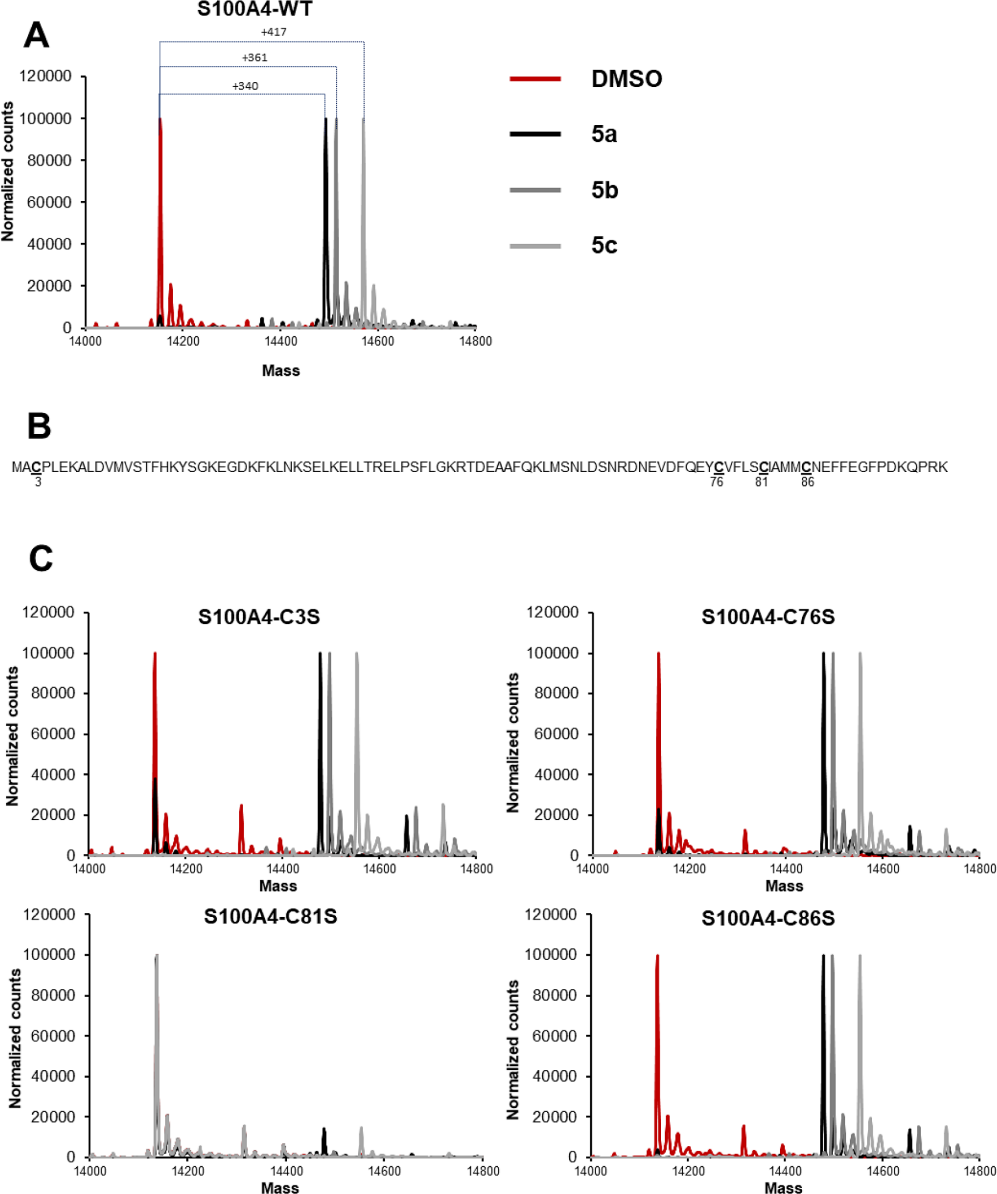
(A) Spectra of S100A4 and (B and C) S100A4-cysteine to serine mutant analysis by mass spectrometry after incubation with DMSO (red), **5a** (black), **5b** (gray), and **5c** (light gray).

To further characterize the mechanism of inhibition of additional covalent compounds, protein labeling was performed by incubating S100A4 with **5a**, **5c**, and **5b** in parallel, followed by mass-spectrometry analysis. In each experiment, spectra indicated single shifts of the S100A4 mass, showing that the protein is covalently modified by a single addition of one molecule (Figure 4A). As seen in previous labeling experiments, the shift of the protein mass corresponds to the addition of the molecule with the loss of a chlorine.

To determine the modification site of the inhibitor, we used site-directed mutagenesis to generate four cysteine mutants of S100A4, where cysteine 3, 76, 81, or 86 was replaced by a serine (Figure 4B). Protein labeling experiments performed with S100A4-C3S, S100A4-C76S, and S100A4-C86S showed a similar pattern to the wild-type protein labeling, whereas S100A4-C81S was resistant to protein modification by all three phenylproline inhibitors (Figure 4C). This experiment shows that **5a**, **5c**, and **5b** covalently inhibit S100A4 by reacting specifically with cysteine 81.

### Kinetics and Robustness of S100A4 Inhibition by Phenylproline Derivatives

**Figure 5:**
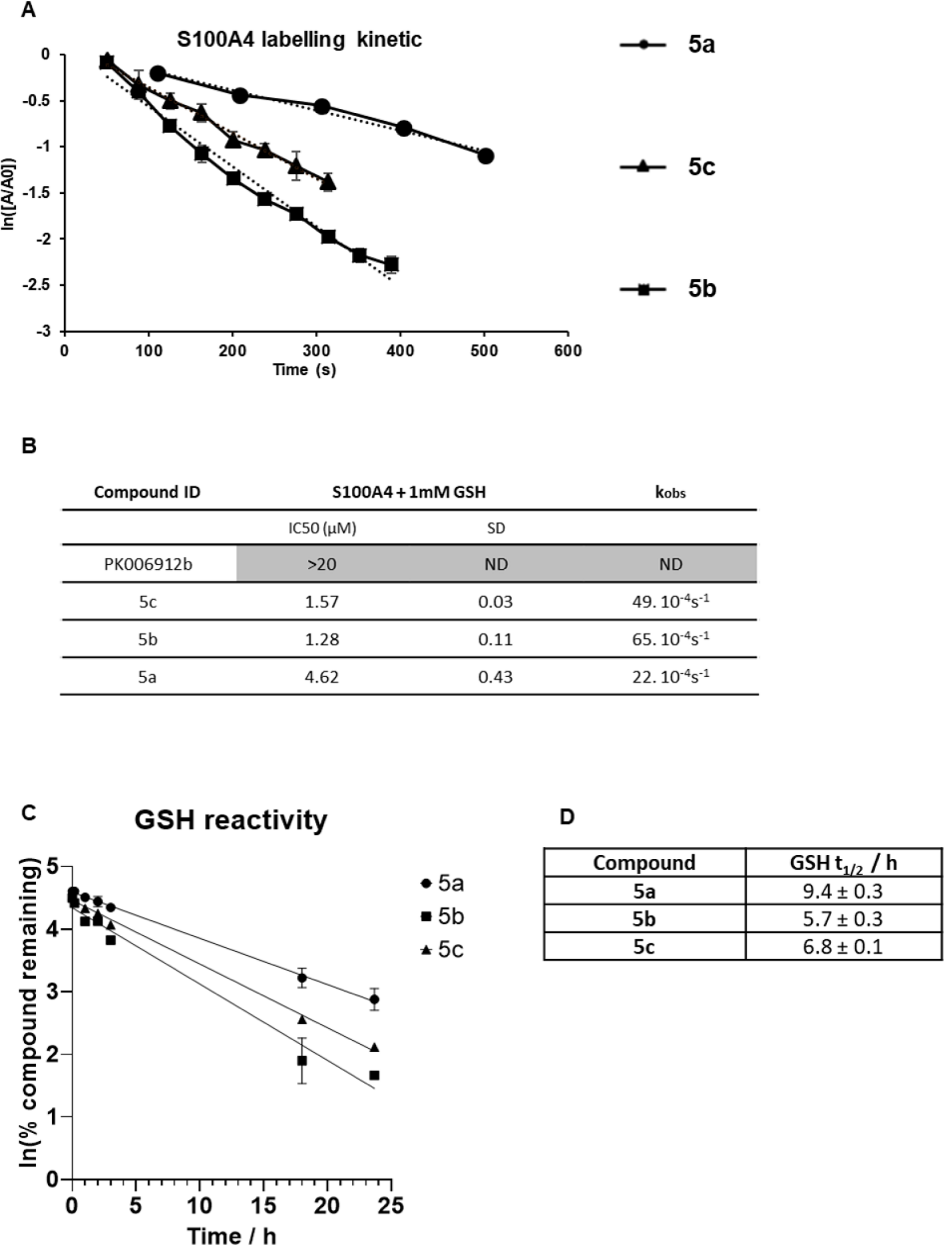
(A) Time course of S100A4 labeling (20 μM) by phenylproline derivative inhibitors (100 μM) for rate determination of their activity. (B) The robustness of their activity has been tested in an HTRF assay for IC_50_ determination in the presence of glutathione, mimicking physiological conditions. (C) Compound reactivity with glutathione has been measured by HPLC to determine the half life of inactivation (D).

In order to determine the kinetics of S100A4 inhibition by phenylproline derivatives, we performed a time course of the detection of unmodified S100A4 (1 μM) against each covalent inhibitor (100 μM) in the presence of calcium. Each experiment was done in triplicate using a RapidFire MS system. The rate of the reaction was determined as −*k*_obs_ = ln([*A*]/[*A*_0_]). Comparison of *k*_obs_ values confirms the high potency of **5b** and **5c** as S100A4 inhibitors with a *k*_obs_ of 65 × 10^–4^ s^–1^ and 49 × 10^–4^ s^–1^, respectively, compared to **5a** with a *k*_obs_ of 22 × 10^–4^ s^–1^ (Figure 5A). These results also correlate with the inhibitor activity previously measured in IC_50_ experiments (Figure 5B), with more potent inhibitors showing faster reaction with cysteine 81.

To measure the robustness of S100A4 inhibition by phenylproline inhibitors and to test whether they would be applicable under physiological conditions, we tested their activities in the presence of glutathione (GSH). GSH is the most abundant low molecular weight thiol compound in the cell. It protects cells from oxidative damage and the toxicity of xenobiotic electrophiles and maintains redox homeostasis.^43^ We speculated that a robust and specific reaction of inhibitors would not be drastically affected by competitive cysteines in the reaction. We performed IC_50_ determination in an HTRF protein–peptide displacement assay in the presence of 1 mM of GSH, a concentration that mimics the amount of glutathione found in the cytoplasm. The results show that PK006912b (**1**) which was highly reactive under previous conditions was no longer active in the presence of GSH, with an IC_50_ over 20 μM (Figure 5B). Phenylproline compounds show a slightly reduced activity against S100A4 with an increase of IC_50_ by a factor of 3 to 4. Under these conditions, phenylproline derivatives **5c** and **5b** still inhibit S100A4 in a micromolar range of concentrations, showing that they are potent and specific inhibitors. To understand how glutathione affects phenylproline inhibitor potency to inhibit S100A4 protein, the inhibitory effect of glutathione has been measured directly by mixing compounds at 50 μM with glutathione at 1 mM and by measuring GSH adducts on S100A4 inhibitors by LCMS. The time course of GSH adduct formation showed that the reactions take several days to be completed (Figure 5C) with calculated *t*_1/2_ of 9.4, 5.7, and 6.8 h for **5a**, **5b**, and **5c** respectively (Figure 5D). These experiments showed that the phenylproline derivatives react preferentially with S100A4 rather than the nonspecific electrophile GSH.

### Phenylproline Derivatives Inhibit S100A4-NMII Interaction in a Cell-Based Assay

**Figure 6:**
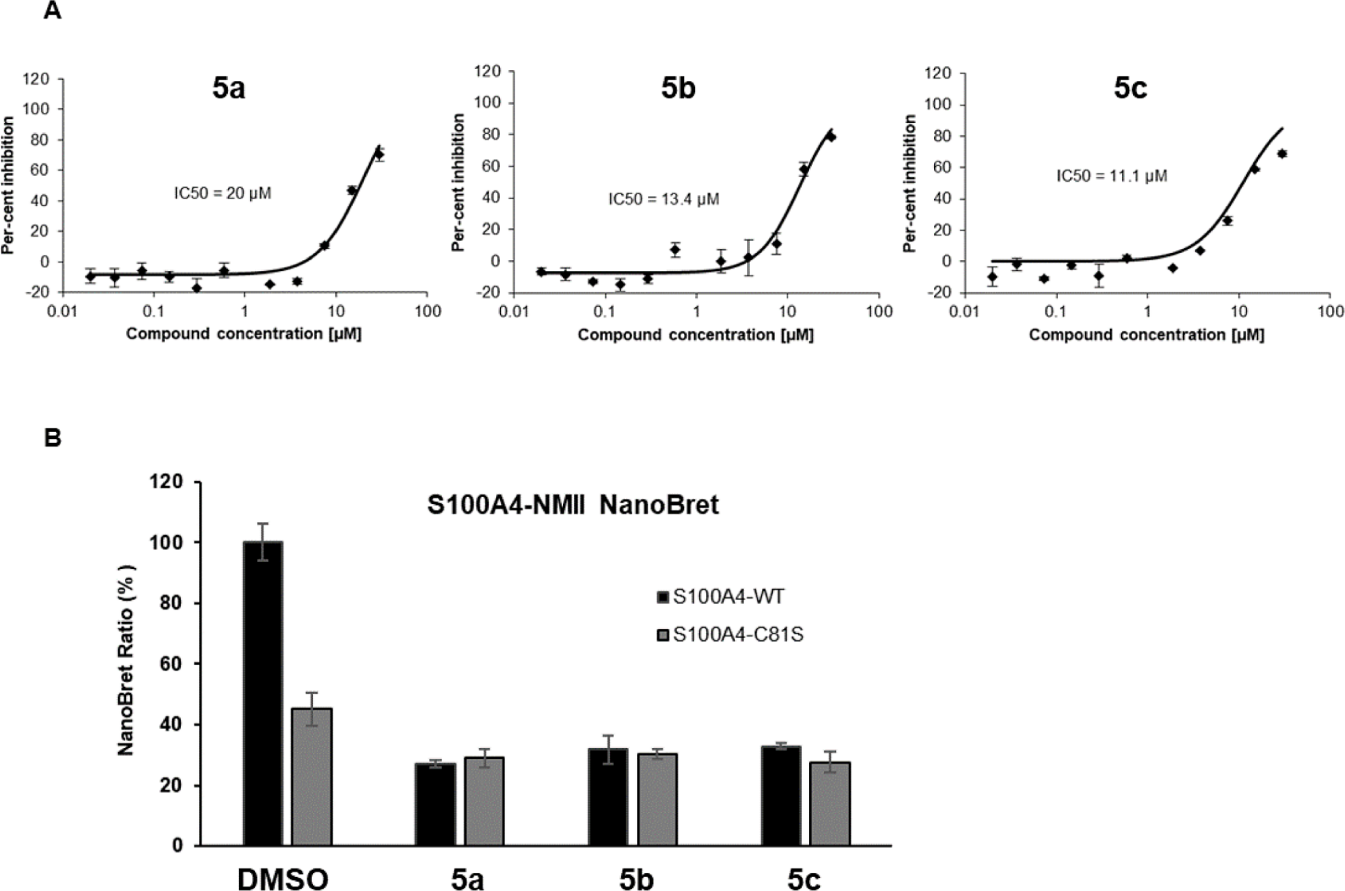
S100A4-NMII interaction inhibitions were measured in IC_50_ NanoBRET experiments (A) and in a single-dose assay in parallel with S100A4-WT and S100A4-C81S (B).

To address whether phenylproline derivatives inhibit the S100A4-NMII complex under physiological conditions, we tested their activities in cell-based assays. First, we performed IC_50_ measurements of protein–protein interaction in the presence of inhibitors using NanoBret technology. Briefly, S100A4-HaloTag and NMII-NanoLuc were expressed in 293HEK cells to allow signal detection from donor (NanoLuc) and acceptor (HaLoTag) proximity upon S100A4-NMII interaction. This protocol resulted in a strong NanoBret signal under control conditions. Testing inhibitors in 11-point curves at a top concentration of 30 μM, and incubating with cells for 24 h, the NanoBret signal dropped in a dose-dependent way (Figure 6A). **5a** showed an IC_50_ of 20 μM, and **5b** showed an IC_50_ of 13.4, and **5c** showed an IC_50_ of 11.1 μM. In order to validate the specificity of the NanoBret interaction and the specificity of the inhibitors, we performed the same experiment by replacing the S100A4 protein with its C81S mutant. We hypothesized that replacing the cysteine targeted by inhibitors would drastically reduce the NanoBret signal in untreated cells, as C81 is on the interface of S100A4-NMII interactions. Additionally, using this mutant would not show any effect of the inhibitors on treated cells as they would be deprived of their target. The S100A4-NMII NanoBret experiment was performed in parallel with S100A4 WT and C81S in the presence of 30 μM inhibitors, a concentration that showed the maximum inhibition in the previous experiment. As expected, S100A4-C81S-NMII showed a reduction of the NanoBret signal compared to S100A4-WT-NMII in DMSO-treated cells (Figure 6B). Results from treated cells showed a similar decrease in the NanoBret signal for both S100 WT and C81S. Inhibitors had a very limited effect on S100A4-C81S as treated cells already showed less than 65% of the untreated signal.

### Effect of Phenylproline Derivative Inhibitors on Cell Growth and Migration

**Figure 7:**
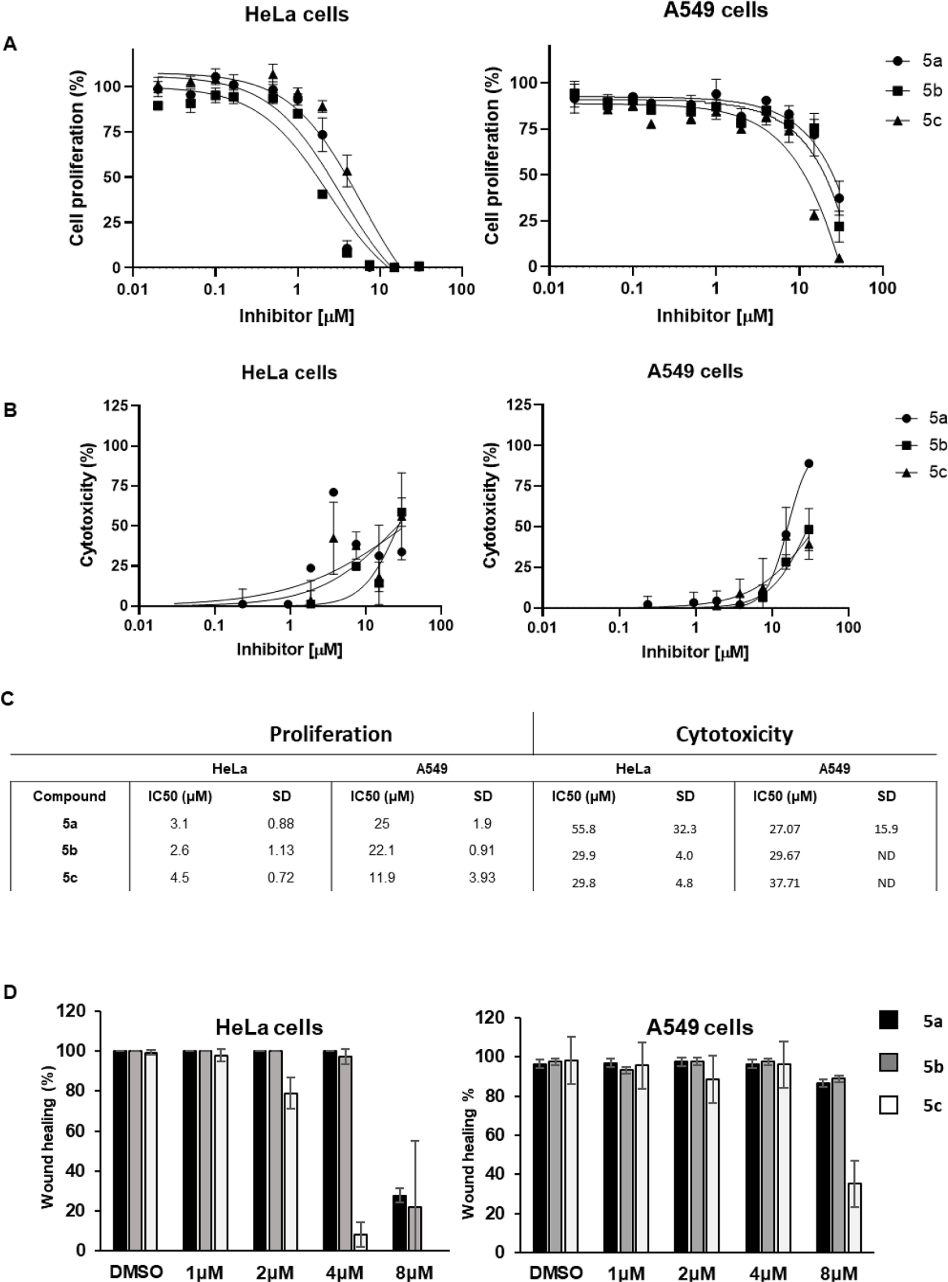
Cell proliferation (A) and cell toxicity (B) assays have been performed on HeLa and A549 treated cells for EC50 determination (C). Cell migration in the presence of an increasing concentrations of inhibitors (D).

To further characterize the potency of the phenylproline derivatives on cancer cells, their effect on proliferation has been measured on HeLa and A549 cell lines, which are known to express S100A4.^44,45^ A cell proliferation assay was performed by cultivating cells for 48 h in complete media with various concentrations of inhibitors, up to 30 μM. At the end of the treatment, luminescence was measured after the addition of the CellTiter-Glo reagent (Figure 7A). Compounds showed fractional toxicity around 50% at the highest concentration tested (Figure 7B,C).

We next investigated whether phenylproline derivatives would inhibit S100A4 activity by affecting cell migration. HeLa and A549 cells were challenged for a cell migration assay in the presence of phenylproline derivatives. A monolayer of cells was scratched and treated with various concentrations of inhibitors for 36 h. Cell migration was monitored automatically using the Incucyte live-cell imager by imaging cells every 2 h. Our results showed that **5c** drastically reduced wound healing at 4 μM, and that **5a** and **5b** at 8 μM in Hela cells (Figure 7D). A549 cells were more resistant to phenylproline derivatives as they showed a moderate effect on cell migration with only 40% of wound healing for **5c** at 8 μM. Taken together, these results showed that phenylproline derivatives, especially **5c**, are potent inhibitors of cell migration and proliferation, which mostly correlate with S100A4-NMII interaction inhibition and less with the general cell toxicity.

**5a**, **5b**, and **5c** were assessed in in vitro ADME assays to compare their aqueous solubility, microsomal stability (MLM), and cell permeability (MDCK-MDR1) (Table S5). On balance, **5b** demonstrated a superior profile consistent with its properties: good solubility and good permeability without efflux by the P-glycoprotein (P-gp) transporter as measured by transit performance in the MDCK-MDR1 cell line. **5a** also had a satisfactory combination of ADME results, whereas **5c** suffered from moderate MLM stability, probably due to its higher lipophilicity.

### X-ray Crystal Structure of Labeled S100A4 Proteins

To better understand the effect of phenylproline derivatives on S100A4, we investigated how they modify its protein structure. We questioned whether they would disrupt the S100A4 dimer or make the S100A4 interacting domain bulky enough to impair NMII interaction. To answer this question, we performed protein crystallography on S100A4 labeled proteins. S100A4 was purified and treated with phenylproline derivatives to reach nearly 100% protein labeling. Then, the protein was concentrated to 2 mM and seeded for crystallization. S100A4-**5a** crystallizes in 8% PEG4000, 0.1 M acetate pH 4.5, and forms crystals that reach their final size after 7 days. S100A4–5b crystallizes in 25% ethylene glycol, and crystals reach their final size after 10 days. S100A4–5c crystallizes under various tested conditions and forms crystals with various sizes and shapes; crystals from each set of conditions were harvested and tested for X-ray analysis.

**Figure 8:**
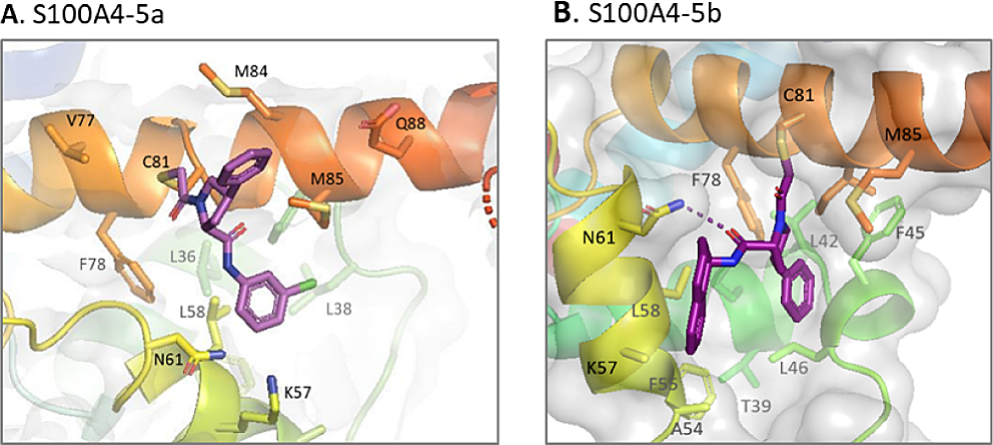
(A) Structure of S100A4 labeled with **5a** (7PSP) and (B) **5b** (7PSQ). The crystal structures were solved using 3C1 V as the starting point.

Except for S100A4–5c crystals, which did not show any diffraction, X-ray data were collected, and S100A4-labeled protein structures were solved using molecular replacement. The S100A4-**5a** structure was solved at a resolution of 2.7 Å with R-free and R-work values of 0.27 and 0.32 respectively (Table S6). The S100A4-**5a** structure (PDB ID: 7PSP) shows a calcium-bound monomer harboring the covalent inhibitor oriented toward the outside of the protein (Figure 8A). The general structure showed a ligand packing that did not allow us to conclude whether the labeled protein was in the monomeric or dimeric form under these conditions. The data confirm the modification of C81 and show an interaction between the chlorophenyl group of **5a** and the methionine 84 of the same S100A4 subunit. The phenyl group of the inhibitor is embedded in the hydrophobic region of the second subunit, surrounded by F45, F55, L46, and L58, resulting in the disruption of the S100A4 dimer and the ligand packing seen in the crystal structure.

A S100A4-**5b** structure (PDB ID: 7PSQ) was solved at a resolution of 1.9 Å with R-free and R-work values of 0.23 and 0.25 respectively. It showed S100A4–5b as a calcium-bound dimer containing one covalent inhibitor bound to C81 of one subunit (Figure 8B). The proline and the phenyl groups of **5b** are oriented in the hydrophobic pocket of the same subunit of S100A4 helices 2, 3, and 4, surrounded by L38, F45, L46, F78, and I82.

## Discussion

In this study, we developed a protein–peptide displacement assay suitable for new S100 protein inhibitor discovery. We tested two readouts for this assay, AlphaScreen and HTRF detection systems, both adapted for a 384 well-plate format. They showed robust signal for their application in high-throughput screening. The screening of our internal library of small molecules, performed in the AlphaScreen format, identified BAY11–7082 as an inhibitor of S100A4. Detailed mutagenesis and mass spectroscopy revealed the covalent nature of inhibition at a single site on the protein interaction surface of S100A4. BAY11–7082 and its analogues are known nonspecific inhibitors of multiple unrelated proteins and are unsuitable as a starting point for drug discovery. The paucity of high-quality noncovalent hits in our screening efforts is not surprising. Protein–protein interactions are notoriously difficult and challenging targets due to shallow binding sites and large interaction surfaces.^46^ Additionally, the choice of TRTK-12 as a common interacting peptide for S100A2, S100A4, S100A5, and S100B might have provided us with specific and nonover reactive hits.

One promising approach that has gained popularity recently is to focus on covalent compounds targeting critical amino acids in the critical spots.^22^ Therefore, we decided to focus our efforts on potential covalent ligands and developed a more specific and robust TR-FRET assay.

This approach led to the discovery of phenylproline derivatives as promising S100A4 inhibitors. They covalently inhibit S100A4 in vitro within a submicromolar range of IC_50_s by labeling cysteine 81. This inhibition is specific as it does not show substantial activity on S100A11, which also contains cysteine at the same position. Additionally, inhibitions are only mildly affected in vitro by the presence of a high concentration of glutathione with an increase of observed IC_50_ by 3–4 folds. This is highly encouraging considering the reductive environment in cells. Interestingly, at the same 1 mM GSH concentration, the activity of the more reactive PK006912a (**1**) compounds has been completely blocked. This relatively low reactivity of the chloroacetamide group allowed novel inhibitors to retain potency in cell-based experiments, as confirmed in NanoBret experiments. The cell permeability and on-target activity are reflected in the phenotypic observations. Cancer cells treated with novel phenylproline inhibitors showed a reduction in cell proliferation and migration, which is very consistent with the inhibition of S100A4-NMII interactions and its downstream signaling. Further investigations in well-characterized cancer cell lines with different S100A4 expression levels would be needed to confirm this activity and are the subject of future work.

The compounds discovered in this work are the most potent S100A4 inhibitors reported to date, as measured by direct biophysical and biochemical methods. They, however, still need improvement in terms of potency and physicochemical properties. This should be helped by structure-based drug design methods, using new crystal structures. The structure determination of the S100A4-NMII complex showed the central role of S100A4-C81 in its interaction with NMII.^22,47^ In the calcium-bound S100A4 dimer, the helices H4 and H4’ from each S100A4 subunit form an antiparallel structure that exposes C81 and C81’ on the NMII binding site. Hydrophobic contacts have been reported between C81 and T1906, A1907, and M1910 of NMII and between C81’ and V1914, L1917, and K1918.^22^ The labeling of both C81 and C81’ by phenylproline compounds generates a bulky structure that prevents NMII from interacting with its binding site. Furthermore, the proline part of the inhibitor fills the S100A4 hydrophobic regions necessary for NMII interactions. Thus, our cocrystal structures open avenues for the rational design of more potent inhibitors based on the current or alternative scaffolds, as well as potential vectors for various PROTAC expansions.

Overall, our results show that the covalent phenylproline inhibitors are effective and specific inhibitors of the S100A4-NMII interactions. Further investigation and optimization of **5b**, which has the best solubility and structural data, could produce lead compounds that would help to better understand the S100A4-NMII signaling cascade and could lead to new therapeutics against metastatic cancers.

## Experimental Procedures

### Reagents

TRTK-12-Biot (Biotin-RRQLPVTRTKIDWNKILS), MPN-Biot (RKLQRELEDATETADAMNREVSSLKNKLRRGGK-biotin), and Annexin2-Biot (Ac-STVHEILSKLSLEGDHSTGGGK(biotin)) peptides were from LifeTein LCC (Hillsborough, NJ). BAY 11–7082 was purchased from Millipore Sigma (USA). Phenylproline derivatives were synthesized in-house and were >95% pure by HPLC analysis (see the Supporting Information).

### Constructs and Protein Purification

S100 protein sequences were cloned in the pNIC28-Bsa4 vector (Addgene, Watertown, MA) using the LIC method as described previously.^48^ Constructs allow for inducible expression of an N-terminal TEV-cleavable His-tag protein in Rosetta (DE3) cells. The four cysteine-to-serine mutants of S100A4 were made by site-directed mutagenesis (Quick change II, Agilent Technologies, Santa Clara, CA) using the following primers: tccaatccatggcgagccctctggagaag and cttctccagagggctcgccatggattgga for S100A4-C3S, ggacttccaagagtacagtgtcctgtcctg and caggacaggaagacactgtactcttggaagtcc for S100A4A-C76S, ctgtgtcttcctgtccagcatcgccatgatgtg and cacatcatggcgatgctggacaggaagacacag for S100A4A-C81S, tcctgccatgatgagtaacgaattctttgaag and cttcaaagaattcgttactcatcatggcgatgcagga for S100A4A-C86S.

Bacteria were grown in the TB medium supplemented with 50 μg/mL kanamycin and 34 μg/mL chloramphenicol at 37 °C under agitation until OD ∼ 1. 1 mM IPTG was added to the medium to induce protein expression, the temperature was turned down to 18 °C, and cells were incubated overnight. Bacteria were harvested by centrifugation at 4 °C and suspended in purification buffer (500 mM NaCl, 50 mM HEPES pH 7.5, 0.5 mM TCEP) containing EDTA-free protease inhibitor cocktail (Roche, Basel, Switzerland) and 10 mM imidazole. Bacteria were sonicated for 15 min on ice, and the cell lysate was clarified by centrifugation at 14 000 rpm (JLA-16 rotor in Avanti J-26S XP centrifuge; Beckman Coulter, Atlanta, GA) for 1 h and passed through a NiNTA column (HisTrap, GE Healthcare Lifesciences, Buckinghamshire, UK). The resin was washed four times with buffer, and protein was eluted using the purification buffer containing 500 mM imidazole. The eluate supplemented with 1 mM EDTA was separated on a size exclusion column (GE Superdex 75 column) using an ÄKTAxpress system (GE Healthcare Lifesciences), and fractions were analyzed by SDS-PAGE. Fractions of the protein of interest were pooled and concentrated by centrifugation (Amicon concentrators 10 kDa, Sigmaaldrich, St. Louis, MO) until the desired concentration reading *A*_280_ using a Nanodrop ND-1000 spectrophotometer (Thermofisher, Waltham, MA). Protein was analyzed by mass-spectroscopy to confirm its expected molecular weight and stored at −80 °C.

### Single Shot Assay

A 4 989 compound library was screened using an Alpha-Screen assay. 6His-Tag-S100A2, -S100A4, -S100A5, and -S100B were used in combination with TRTK-Biotin peptide under conditions described previously. Compounds were dispensed at a single dose of 50 μM (final assay concentration) in duplicate onto 384-well ProxiPlates using the Echo 520 liquid dispenser (Labcyte). Twelve wells per plate were used as solvent controls and as a reference for inhibition calculation, which was calculated as inhibition (%) = (1 – (*F*_sample_– *F*_background_)/(*F*_solvent_ – *F*_background_)) × 100

### IC_50_ Determination Assay

The best hits from the single-shot experiment were tested for IC_50_ determination using the HTRF assay as described above. Compounds were dispensed in duplicate onto 384-well Proxiplates at 15 different concentrations using a dilution factor of 2. The percentage of inhibition was calculated as described above and was used for IC_50_ determination.

### Protein Labeling Detection by Mass Spectrometry Analysis

Proteins of interest were diluted to 10 μM in 100 mM NaCl, 25 mM HEPES pH 7.5, and 1 mM CaCl_2_ buffer in the presence of inhibitors, allowing a protein–compound ratio of 1:10. The mixture was incubated for 1 h at room temperature in the dark and analyzed by mass spectrometry using a 1290 Infinity coupled to a 6530 Accurate-Mass Q-TOF LC/MS (Agilent Technologies). Results were analyzed using the Qualitative Analysis MassHunter Acquisition Data and the Bioconfirm software.

For kinetic determination of S100A4 inhibition, 1 μM S100A4 was mixed with a 100 μM covalent inhibitor in 50 mM MES, pH 7.0, and 1 mM CaCl_2_ buffer. The mixture was analyzed over time using the RapidFire MS system (Agilent Technologies).

### GSH Reactivity Assay

50 μM of each compound was incubated at room temperature with 1 mM GSH in 50 mM HEPES, 20 mM NaCl, pH 7.5, and 1.3% DMSO. At each time point, an aliquot was taken and analyzed by LCMS. The percentage of the compound remaining was determined by comparing the area of the peaks of the compound and the GSH adduct. The natural logarithm of the results was fitted to a linear regression, and *t*_1/2_ was calculated as *t*_1/2_ = ln(2)/–slope.

### Biophysicochemical Property Measurement of the Inhibitor

Selected compounds were screened for aqueous solubility in PBS (pH 7.4), metabolic stability in mouse liver microsomes (MLM) as a measure of clearance, Log *D*, and permeability. ADME studies reported in this work were independently performed by WuXi AppTec (Shanghai, China). Raw data are provided upon request.

### Crystallization of Labeled S100A4 and X-ray Analysis

S100A4 protein was diluted to 70 μM in 100 mM NaCl, 25 mM HEPES pH 7.5, 1 mM CaCl_2_, and 0.5 mM TCEP containing inhibitors at 90 μM, and incubated for 1 h at room temperature. Protein labeling was confirmed by mass spectrometry by analyzing an aliquot of the mixture. Labeled proteins were concentrated using Amicon cell concentrators (3 kDa), washed twice with crystallization buffer (100 mM NaCl, 50 mM MES, 2 mM CaCl_2_, and 0.5 mM TCEP) and concentrated again until the protein concentration reached ∼2 mM. Labeled S100A4 and precipitant mixes were dispensed into sitting-drop plates using the Mosquito liquid dispenser (Sptlabtech, England). The drop volume used was 300 nl, and the reservoir volume was 50 μL. Crystals appeared after two to 7 days and reached their final size of ∼80 μm after one to 2 weeks. Crystals were soaked with 20% ethylene glycol as a cryoprotectant, fished, and kept in liquid nitrogen until their X-ray data collection at Diamond Light Source. X-ray data were analyzed using Phoenix, WinCoot, and CCP4 softwares.

### NanoBret Binding Assay

S100A4 and NMII Nanobret fusion constructs were generated by cloning coding sequences into linearized dedicated vectors by using EcoRV and XbaI restriction sites. Assay development was done according to the NanoBRet-NanoGlo detection system manufacturer’s recommendations (Promega) by testing each combination of *N*- and *C*-termini of HaloTag and NanoLuc fusion constructs. 293HEK cells were seeded on 6-well plates in complete DMEM media and transfected 6 h later with S100A4-NanoLuc (1.8 μg) and NMII-HaloTag (0.2 μg) constructs using the JetPrime transfection reagent (Polyplus Transfection, Strasbourg, France). After an overnight incubation, cells were resuspended in phenol-red-free, 4% serum media in the presence of the HaloTag NanoBret 618 ligand or DMSO for control. They were transferred to a white 384-well plate containing inhibitors and left for 24 h in a cell culture incubator. NanoBret NanoGlo substrates were added to cells prior to reading the plate with the Pherastar FSX plate reader. The raw Nanobret ratio was calculated by dividing the acceptor emission value (618 nm) by the donor emission value (460 nm) for each sample. The mean NanoBret data were calculated by subtracting the background ratio (no acceptor control) from the sample ratio.

### Cell Proliferation and Cytotoxicity Assay

The day before, cells were seeded in a white, cell-culture treated 384-well plates in complete high-glucose DMEM to reach a confluency of 25% on the day of the experiment. The next day, the medium was replaced by complete, high-glucose, phenol-red-free medium, and cells were incubated for 48 h in the presence of an inhibitor or DMSO as a control. Aliquots of the supernatant were harvested, diluted 100 times in 200 mM Tris-HCl, pH 7.3, 10% glycerol, and 1% BSA buffer, and used for cyctotoxicity measurement by using the LDH-Glo cytotoxicity assay kit (Promega). In parallel, the CellTiter-Glo (Promega) reagent was added to the cells to measure cell proliferation. Both experiments were done according to the manufacturer’s recommendations, and the luminescence was quantified using the FSX plate reader (Pherastar).

### Cell Migration Assay

The day before the experiment, cells were seeded in a black, clear-bottom, collagen-coated 96-well plate in complete high-glucose DMEM to reach nearly full confluency the next day. On the day of the experiment, a wound in the cell layer was made, and the media were replaced with media containing phenylproline derivatives. Cells were incubated for 36 h in an Incucyte cell-imager (Sartorius, Germany), allowing for automated cell imaging every 2 h. The analysis was done by measuring the wound area using ImageJ and plugging the wound healing tool.

## Supporting Information Available

Experimental details and results of AlphaScreen and HTRF-screening, compound synthesis and physicochemical properties, X-ray data collection, and refinement statistics (PDF)
Molecular formula strings (CSV)
