# Supplementary material for: Covalent Inhibitors of S100A4 Block the Formation of a Pro-Metastasis Non-Muscle Myosin 2A Complex
Source: J Med Chem. 2024 Oct 19;67(21):18943–56. doi: 10.1021/acs.jmedchem.4c01320 (PMC11571109; doi:10.1021/acs.jmedchem.4c01320)
Supplement: Supplementary file 1 — jm4c01320_si_001.pdf [file jm4c01320_si_001.pdf]

Supporting information

Covalent inhibitors of S100A4 block formation of pro-metastasis Non-Muscle Myosin 2A complex

Charline Giroud[†], Tamas Szommer[†], Carmen Coxon[†,‡], Octovia Monteiro[†,§], Thomas Grimes[† ⱡ], Tryfon Zarganes-Tzitzikas[† ⱡ], Thomas Christott[†], James Bennett[†,□], Karly Buchan[†], Paul E. Brennan[†], Oleg Fedorov[†,⁑]

[†] *Centre for Medicines Discovery, Nuffield Department of Medicine, NDM Research building, Old Road Campus, Oxford OX3 7FZ, UK.*

[ⱡ] *Alzheimer's Research UK Oxford Drug Discovery Institute, NDM Research Building, Old Road Campus, Oxford OX3 7FZ, UK*

[‡] *Current location: MHRA Laboratories, South Mimms, Potters Bar EN6 3QG, UK.*

[§] *Current location: Hexagon Bio, 1490 O'Brien Dr, Menlo Park, CA 94025, United States.*

[□] *Current location: Exscientia, Oxford Science Park, The Schrödinger Building, Oxford OX4 4GE, UK.*

[⁑] *Lead contact.*

Contents

Covalent inhibitors of S100A4 block formation of pro-metastasis Non-Muscle Myosin 2A complex ..S1

Peptide displacement assay development..S2

Figure S1: S100 peptide-displacement assay development for Alphascreen high-throughput screening...S3

Table S1: Experimental conditions used for S100 Alphascreen high-throughput screening. FAC: final assay concentration. S3

Figure S2: Scatter-plot of S100-TRTK High throughput screening done in Alphascreen optimized in 384-well plates. Compounds were tested in duplicate at a single dose of 50 µM. S4

Figure S3: S100 peptide-displacement assay development for HTRF high-throughput screening. S6

Table S2: Experimental conditions of peptide-displacement assay for S100A4-NMIIA and S100A11-AnII inhibition using HTRF readout. FAC: final assay concentration.............................S6

Table S3: Screening results of HTRF single-shot experiment performed on S100A4 and S100A11 for testing NU000846 (5) related compounds. S7

Compound synthesis...S8

Scheme 1. General synthetic route S8

General procedure for the synthesis of intermediates 3a-g. S9
General procedure for the synthesis of intermediates 4a-g. S9
General procedure of products 5a-g S9
Table S4: Compound IDs and SMILES S30
Table S5: Physicochemical and molecular properties S31
Crystallography S32
Table S6: Data collection and refinement statistics. S32

Peptide displacement assay development

This experiment is based on a complex disruption of 6His-tag protein and a biotinylated peptide by addition of an inhibitor. The conditions for an optimal protein-peptide complex formation were done by mixing a serial dilution of the 6His-tag protein with a serial dilution of biotinylated peptide, allowing final concentrations of each component from 3.2 µM to 0.2 nM. Assay was done in 384-well ProxiPlates (Labcyte, San Jose, CA) in 100 mM NaCl, 25 mM HEPES pH 7.5, 1 mM $CaCl_2$, 0.1% BSA, 0.05% Tween-20 buffer. After one hour of incubation at room temperature, detection reagents were added to the mix according to the manufacturer recommendations, AlphaScreen Histidine (Nickel Chelate) Detection Kit (PerkinElmer, Waltham, MA) for Alpha-Screen assay, or SA-XL665 (1:500, PerkinElmer) and anti-6His-EU (1:10,000, PerkinElmer) for HTRF assay. After 2 hours of incubation at room temperature in the dark, plate fluorescence was read using Pherastar FSX plate reader (BMG Labtech, Ortenberg, Germany) with the appropriate AlphaScreen or HTRF optic modules. The optimal protein and peptide concentrations were chosen to have the best signal to background in the lowest concentration range to avoid the saturation of the signal.

Figure S1: S100 peptide-displacement assay development for Alphascreen high-throughput screening.

S100A2 (nM)	TRTK (nM)															
	3200	1600	800	400	200	100	50	25	12.5	6.25	3.13	1.56	0.78	0.39	0.20	0
3200	685102	583091	521664	172254	70908	35910	31331	32072	30039	31293	32338	26961	34219	27512	30001	29754
1600	766080	663613	660364	458489	259084	61902	45220	40261	37753	39881	34485	33155	38342	34390	34580	35511
800	713925	673398	650446	547960	429761	142386	75734	45866	40451	35739	34751	33858	36271	33022	35207	31198
400	731728	691353	679022	603896	508535	263302	142196	59375	47975	46398	39748	34390	36841	34770	35207	36233
200	691809	648375	649515	607924	576935	459857	374794	152988	81206	50445	35074	36024	43928	36461	35131	35055
100	698573	669047	695324	705622	583072	567492	477451	327028	176852	59109	49419	42845	43738	36062	39900	39634
50	706268	657799	662644	640566	607221	584421	558087	481992	389101	131043	68115	42161	40166	34390	37107	34732
25	701841	710562	695381	673626	674614	658635	622174	604143	545984	262922	107787	41724	38893	33269	35055	31730
12.5	599621	595764	597702	599583	576080	570171	560272	576802	530917	408386	207252	47785	33915	28671	24700	24757
6.25	501334	530727	469300	533957	485279	407626	508687	525312	495691	344356	203433	39862	26429	22534	18905	20007
3.13	256785	312816	275272	296248	265905	254505	233852	279148	269971	221407	124697	38532	19665	15219	13072	12654
1.56	155078	194351	164692	194807	157757	218918	163590	200279	171836	71953	46607	15656	10773	11647	8436	9994
0.78	10659	47861	32091	49989	29963	60838	41895	56753	42218	22648	15143	9728	6042	4750	6118	6650
0.39	5510	12483	6688	9633	8113	13718	8075	11704	9082	6156	5510	3933	3667	3382	3591	3762
0.20	2641	1691	1178	3762	1159	1748	1900	1539	1710	3059	1501	2527	1197	2185	1235	2166
0	1615	1292	1216	1083	1121	912	874	893	1083	1007	1140	1159	1558	1501	1406	1254

S100A4 (nM)	TRTK (nM)															
	3200	1600	800	400	200	100	50	25	12.5	6.25	3.13	1.56	0.78	0.39	0.20	0
3200	860738	781850	804821	641649	370842	150024	113848	75506	72960	69844	63023	53713	47804	47329	45752	47500
1600	910613	815214	833017	741969	641896	432212	286368	97356	82441	66101	54587	44080	44061	39634	36993	39444
800	882056	806892	826196	763933	654930	547010	413345	133171	99826	67070	61750	45847	44707	41116	41420	37221
400	837900	754680	774478	717915	627342	592116	506692	275044	167276	85576	71174	49799	51110	49191	45106	45733
200	850972	768892	770716	725743	614498	598367	521626	330486	215840	100738	78128	60952	57475	48450	54568	54872
100	603003	658350	687857	625613	441427	495672	474886	358891	267520	144571	104101	67336	59394	53865	53542	51395
50	623162	453625	462631	449559	256576	348308	369797	281162	237956	149986	77349	70528	66329	60078	58919	63023
25	45733	478420	61921	462802	225625	330258	367422	322677	320055	185136	148675	78888	66082	63536	55841	56696
12.5	382090	360582	399361	366700	138263	242250	278103	281846	275424	185630	152152	80484	61294	60439	50635	51984
6.25	4674	6422	8645	13604	8835	12369	17195	28253	34409	58330	72504	59299	49457	45600	37829	38855
3.13	2413	3477	3724	5605	3933	7999	9785	15732	22819	43453	41363	43130	34789	35188	30153	29241
1.56	2318	779	874	1615	1577	3534	4769	6251	8265	15086	20007	23883	20767	20349	17841	16226
0.78	4484	1273	2052	2432	2489	3173	4104	5510	7752	17917	21033	20881	17233	16131	14364	14839
0.39	1159	1235	1007	1007	1178	1824	1311	2622	2698	5149	6042	7923	6669	7733	7201	6726
0.20	1444	950	874	722	722	1330	1140	1691	1805	3515	4503	4655	4845	4522	3591	4826
0	1026	1216	779	1102	1349	1026	950	1292	817	1254	1349	2071	2394	3249	2033	3610

S100B (nM)	TRTK (nM)															
	3200	1600	800	400	200	100	50	25	12.5	6.25	3.13	1.56	0.78	0.39	0.20	0
3200	22287	17157	20653	17195	16834	15352	14763	10526	13110	17138	19703	19038	20938	17575	22116	21261
1600	29773	21622	23921	21280	18943	18088	20121	14307	15409	16112	20045	21907	22420	20881	23427	22857
800	41382	31844	31787	31692	29697	25327	26657	20767	21394	15580	24282	24871	28823	26049	30628	30115
400	49343	44821	40109	39520	38133	37031	37696	30780	29697	21375	27189	29944	36765	32319	42199	40128
200	58672	53048	58805	53086	50749	45011	55233	39501	45429	25251	32034	35568	47500	43852	50787	53029
100	74803	70908	65398	63688	64030	60952	61921	50882	54758	37715	32053	39235	52934	49286	61845	63384
50	87457	84056	82251	73625	80750	68476	86925	71288	75012	48887	35549	40109	51034	54207	72219	72029
25	114019	105887	116147	113525	110428	108490	115862	94259	111986	83353	49210	30400	31046	44764	50407	64068
12.5	155743	148675	165794	159885	162678	157890	172615	146699	175769	118978	62567	32756	30552	41781	44137	57057
6.25	185687	188328	236094	219526	241623	236246	279072	221122	295849	221065	95494	43890	28766	25346	26961	37677
3.13	218006	243599	326002	328681	355224	370272	416442	363413	433713	310859	99161	43149	25783	20121	20653	31445
1.56	147611	167295	217132	254239	300941	284202	288990	333127	300979	367574	145692	45486	22952	15903	13680	19722
0.78	84170	83049	99465	113734	128763	118940	121904	145635	121087	203604	102733	28405	12711	10583	8588	12711
0.39	33668	32661	40299	45144	47937	52440	48697	60230	52516	68400	93062	28747	9405	5719	4959	6954
0.20	22629	18430	22686	26049	28177	25555	23959	34979	25498	38076	46892	13832	4902	3249	2755	7866
0	2147	1140	969	1558	988	1216	893	1444	2983	1273	1349	2337	2660	2660	3591	4826

S100A5 (nM)	TRTK (nM)															
	3200	1600	800	400	200	100	50	25	12.5	6.25	3.13	1.56	0.78	0.39	0.20	0
3200	832656	737694	770849	613187	501334	228076	115482	58235	59907	59812	67469	70604	75753	66557	77026	78147
1600	861441	771723	786752	701461	664145	415454	251693	90706	77862	69578	79458	74898	88350	83961	89661	93917
800	838185	802180	844113	781717	789184	629242	518909	189620	135318	84474	98021	89072	103740	97147	107958	108319
400	929746	816126	884146	793896	824030	715540	712766	406885	282264	109535	112575	102828	122854	104082	118997	124108
200	854582	815005	873487	784320	850611	755155	773490	579994	495862	168530	124545	111264	121581	114798	123785	127205
100	802655	794124	842973	784643	824847	743546	758613	626525	584041	265753	134843	111055	111321	109345	114000	117914
50	793003	768379	839192	754072	811414	715008	777765	661960	659851	483113	172710	90706	80731	86754	82859	87381
25	586834	612731	649762	612693	660687	607848	619400	568613	585029	512278	421059	103113	59945	57418	48735	55081
12.5	515831	547713	603079	548245	595802	553261	541291	531430	515926	463182	297027	81890	43681	41534	41154	41914
6.25	278407	347966	345724	348365	394820	366434	367517	351424	359024	316236	238336	77520	28025	27360	24339	26505
3.13	171950	237139	262618	231344	251731	250800	223706	227392	216315	160094	77995	38665	19703	21033	16853	19171
1.56	29564	69540	23123	74993	78508	84379	71212	77235	63232	46968	33098	15238	9747	9918	8854	11894
0.78	5605	9785	8987	12882	14535	16207	10963	15884	11742	8664	8816	6232	5472	5947	5928	7790
0.39	1577	1900	2679	2014	1634	2109	2337	2470	1691	2679	2508	2964	2147	3116	2641	4560
0.20	3306	1539	1463	1634	1273	1292	1330	1140	1349	2052	1938	2204	2166	1729	2147	3990
0	1501	1596	1007	494	950	1273	836	741	893	836	1064	1843	1824	2014	1653	6251

Table S1: Experimental conditions used for S100 Alphascreen high-throughput screening. FAC: final assay concentration.

His-Tag Protein	S100A2	S100A4	S100A5	S100B
**Protein FAC (nM)**	12.5	100	50	3.125
**Peptide Description**	Bio-RRQLPVTRTKIDWNKILS			
**Peptide FAC (nM)**	50	200	50	12.5
**Buffer**	100mM NaCl, 25mM HEPES, 0.1% BSA, 0.05% Tween-20, 1mM $CaCl_2$, pH 7.5			
**S/B**	22.6	8.5	13.8	8.9

Figure S2: Scatter-plot of S100-TRTK High throughput screening done in Alphascreen optimized in 384-well plates. Compounds were tested in duplicate at a single dose of 50 μM.

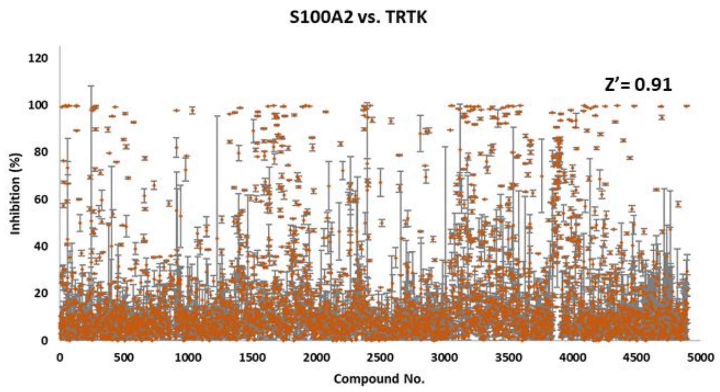
S100A2 vs. TRTK
Z'= 0.91
Inhibition (%)
Compound No.

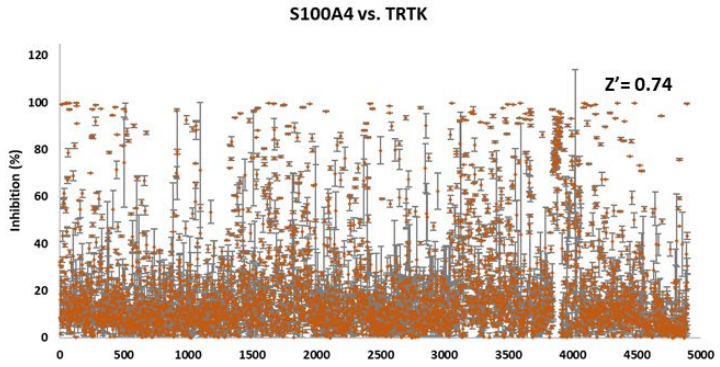
S100A4 vs. TRTK
Z'= 0.74
Inhibition (%)

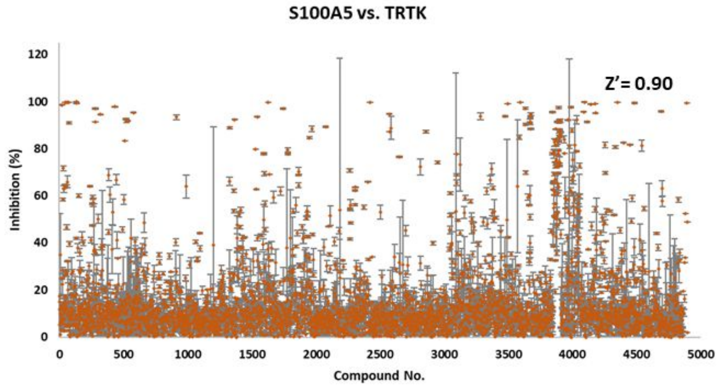
S100A5 vs. TRTK
Z'= 0.90
Inhibition (%)
Compound No.

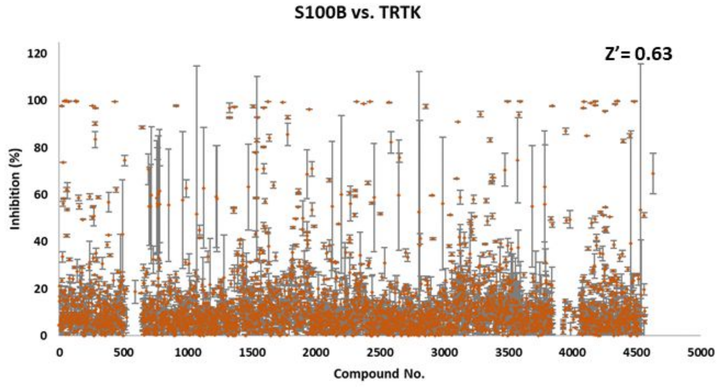
S100B vs. TRTK
Z'= 0.63
Inhibition (%)
Compound No.

Figure S3: S100 peptide-displacement assay development for HTRF high-throughput screening.

S100A4(nM) \ NMII (nM)	3200	1600	800	400	200	100	50	25	12.5	6.25	3.13	1.56	0.78	0.39	0.20	0
3200	442	2230	2567	3398	3089	3664	3681	2067	849	337	211	161	150	134	139	134
1600	364	3077	4053	5183	5340	5806	6002	3540	1464	508	276	177	155	147	145	146
800	314	1865	4961	6914	7383	8115	8059	5134	2653	1008	480	259	178	152	149	141
400	269	928	2863	6483	8345	8966	9046	6513	3727	1575	756	357	217	162	147	139
200	220	722	2111	5610	9108	9654	9318	6824	3952	1792	945	433	253	182	153	136
100	178	497	1301	3367	7508	9857	9365	7418	4652	2374	1145	570	308	194	156	139
50	180	389	768	2281	5722	9196	9438	7059	4662	2281	1230	581	303	197	156	138
25	161	289	569	1404	4070	7582	9128	6689	4050	2287	1090	562	271	192	148	132
12.5	163	259	452	749	2051	5137	7649	6277	3713	1907	920	435	254	179	158	143
6.25	153	222	331	723	1534	4112	5739	4383	2395	1336	664	344	216	155	144	136
3.13	133	185	224	330	695	1668	3488	2539	1644	939	474	268	187	152	145	135
1.56	142	169	211	255	507	990	2059	1500	828	495	308	192	160	142	141	132
0.78	142	157	175	189	280	623	987	743	490	326	216	182	151	135	142	134
0.39	135	150	150	176	213	279	440	382	284	212	168	150	140	144	144	130
0.20	137	144	145	147	171	213	283	232	197	167	149	147	134	134	133	147
0	130	138	142	135	133	142	166	137	137	131	142	133	137	138	132	132

S100A11 (nM) \ AnII (nM)	3200	1600	800	400	200	100	50	25	12.5	6.25	3.13	1.56	0.78	0.39	0.20	0
3200	1215	1260	1193	1071	1089	1035	1012	615	324	270	196	160	142	128	124	113
1600	2351	2579	2465	1922	2075	1662	1608	1242	560	381	227	174	147	129	119	113
800	3633	3834	4197	3166	2945	2379	2407	1614	626	361	197	150	130	122	118	110
400	4165	4875	4871	4007	3602	2797	2756	2450	874	476	245	160	128	120	119	111
200	5227	5743	5603	4742	4132	3401	3330	1990	725	387	189	140	125	115	118	112
100	5691	6376	6281	5319	4546	3563	3335	2997	1095	516	231	154	125	120	110	111
50	5717	6844	6838	6003	5195	3918	3377	3583	1302	595	268	155	127	118	113	111
25	6585	7376	7390	6438	5605	4145	3902	3240	1183	510	269	157	125	116	111	108
12.5	6401	7327	7191	6588	5538	3978	3574	3634	1468	521	265	159	120	115	110	108
6.25	6005	6630	6395	5980	4996	3731	4004	2562	892	400	199	149	118	113	111	110
3.13	4111	4879	4629	4729	3741	2906	2802	2165	849	326	173	135	112	110	110	106
1.56	2656	3195	3038	3140	2584	2033	1932	1868	691	293	170	129	109	108	108	106
0.78	1688	2492	2472	2411	1861	1567	1813	961	411	210	138	114	110	109	107	107
0.39	1377	1682	1771	1949	1486	1304	1304	1111	447	204	136	118	108	109	106	105
0.20	954	1336	1250	1275	1045	987	1386	101	321	162	127	113	107	108	107	109
0	100	101	154	99	103	99	107	101	100	104	106	107	104	107	106	104

Table S2: Experimental conditions of peptide-displacement assay for S100A4-NMIIA and S100A11-AnII inhibition using HTRF readout. FAC: final assay concentration.

His-Tag Protein	S100A4	S100A11
Protein FAC	50 nM	12.5 nM
Peptide	RKLQRELEDATETADAMNREVSSLKNKLRRGGK-Bio	Ac-STVHEILSKLSLEGDHSTGGGK-Bio
Peptide FAC	50 nM	600 nM
Buffer	100mM NaCl, 25mM HEPES, 0.1% BSA, 0.05% Tween-20, 1mM $CaCl_2$, pH 7.5	
Z'	0.94	0.85

Table S3: Screening results of HTRF single-shot experiment performed on S100A4 and S100A11 for testing NU000846 (5) related compounds.

Compound ID	Compound Structure	S100A4		S100A11	
		Inh. (%)	Inh. error (%)	Inh. (%)	Inh. error (%)
NU000008a	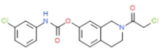	2.78	0.37	1.875	0.565
NU000007a	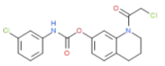	2.925	1.655	3.95	0.04
NU000226a	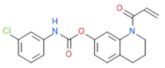	0.29	2.37	1.165	0.465
NU000442a	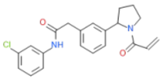	0.555	0.865	0.205	0.085
NU000443a	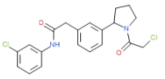	3.495	0.695	3.37	0.79
NU000844a	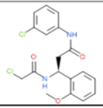	-0.975	0.025	0.375	1.025
NU000845a	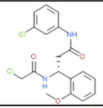	0.51	0.07	0.585	0.195
NU000846a	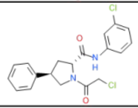	10.58	1.01	2.695	0.595
NU000847a	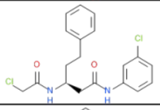	-1.02	0.17	0.92	0.22
NU000848a	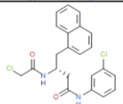	1.13	0.84	0.76	0.44
NU000849a	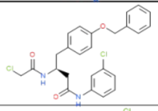	0.29	0.84	-0.55	0.25
NU000850a	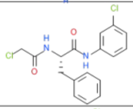	-0.82	0.32	0.55	0.4
NU000857a	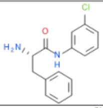	-0.945	1.185	0.05	0.13
NU000946a	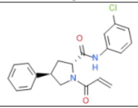	-2.43	1.53	0.43	0.49
NU000947a	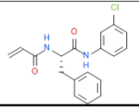	0.925	0.395	-0.735	0.335

Compound synthesis

General procedures. All solvents were purchased from commercial sources and used without purification (HPLC or analytical grade). Anhydrous solvents were purchased from Acros Organics and stored under a nitrogen atmosphere with activated molecular sieves. Standard vacuum line techniques were used, and glassware was oven-dried prior to use. Deionised water was sourced from an Elga DV 25 system. Reactions were monitored by thin layer chromatography (TLC) and/or liquid chromatography-mass spectrometry (LCMS). TLC analysis was performed on commercially prepared aluminium plates coated with 60 F254 silica gel. Plates were visualised using UV light (254 nm). Normal-phase silica gel column chromatography was carried out using a Biotage Isolera One flash column chromatography system with pre-packed SNAP KP-Sil columns. NMR spectra were recorded using a Bruker Avance 400 MHz spectrometer using the deuterated solvent stated. Chemical shifts (δ) quoted in parts per million (ppm) and referenced to the residual solvent peak. Preparative HPLC-MS was carried out using Waters LCMS system (Waters 2767 sample manager, Waters SFO System Fluidics organizer, Waters 2545 binary gradient module, Waters 2489 UV/Vis detector, Waters 2424 ELS detector, Waters SQ detector 2); Column: Kinetex 5 µM EVO C18 column (100 mm × 3.0 mm, 100 Å); eluent A: 93% water, 5% acetonitrile, and 2% of 0.5 M ammonium acetate adjusted to pH 6 with glacial acetic acid, eluent B: 18% water, 80% acetonitrile, and 2% of 0.5 M ammonium acetate adjusted to pH 6 with glacial acetic acid; gradient: 0-0.35 min 5% B, 0.35-1.35 min 5-95% B, 1.35-2.1 min 95% B, 2.1-2.2 min 95-5% B, 2.2-3 min 5% B; flow 2 ml/min; wavelength: 220 nm & 254 nm.

Abbreviations. rt, room temperature; TLC, thin layer chromatography; LCMS, liquid chromatography-mass spectrometry; MS, mass spectrometry; HPLC, high-performance liquid chromatography, NMM, 4-methylmorpholine; HATU, O-(7-Aza-1H-benzotriazol-1-yl)-N,N,N',N'-tetramethyluronium hexafluorophosphate; MeCN, acetonitrile; TFA, trifluoroacetic acid; DCM, dichloromethane; TEA, triethylamine

Scheme 1. General synthetic route

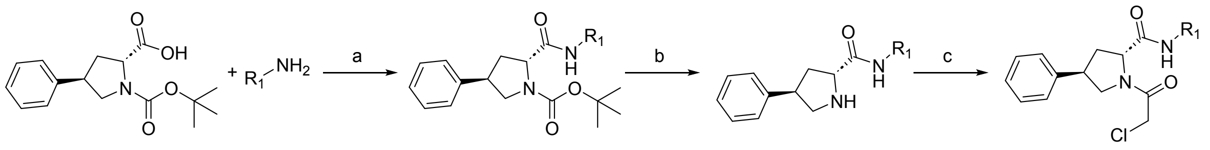

1 **2a-g** **3a-g** **4a-g** **5a-g**

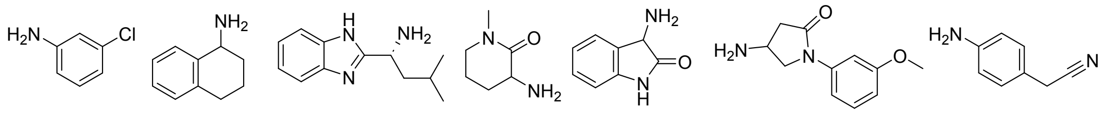

2a **2b** **2c** **2d** **2e** **2f** **2g**

Reagents and conditions: (a) NMM, HATU, MeCN, rt, 16 h (b) TFA, DCM, rt, 16 h (c) TEA, 2-chloroacetyl chloride, DCM, rt, 16 h

General procedure for the synthesis of intermediates 3a-g. (2R,4R)-1-[(tert-butoxy)carbonyl]-4-phenylpyrrolidine-2-carboxylic acid, **1** (1 equiv, 0.343 mmol) was dissolved in dry acetonitrile. After addition of N-methylmorpholine (3 equiv, 0.113 mL, 1.03 mmol) and O-(7-Aza-1H-benzotriazol-1-yl)-N,N,N',N'-tetramethyluronium hexafluorophosphate (1.4 equiv, 182.7 mg, 0.481 mmol) the reaction mixture was stirred at room temperature for 45 min. Amine reagent**, 2a-g** (1.1 equiv, 0.378 mmol) was added to the mixture and further stirred at room temperature for 16 hours while monitoring the progress of the reaction by thin layer chromatography. The solvent was evaporated to dryness and the crude product was purified by flash column chromatography to give **3a-g.**

General procedure for the synthesis of intermediates 4a-g. After purification, intermediate **3a-g** were dissolved in dry dichloromethane (3 mL) and trifluoroacetic acid (16 equiv) was added to the solution. The reaction mixtures were stirred at room temperature for 16 hours while monitoring the progress of the reaction by thin layer chromatography. The solvent was evaporated to dryness. The crude **4a-g** were used in the next reaction step without purification.

General procedure of products 5a-g. The crude **4a-g** were used for the synthesis of product **5a-g. 4a-g** were dissolved in dry dichloromethane then triethylamine (3 equiv) followed by 2-chloroacetyl chloride (1.05 euiv) were added to the solution. The reaction mixtures were stirred at room temperature for 16 hours while monitoring the progress of the reactions by thin layer chromatography. The solvent was evaporated to dryness and the crude **5a-g** were purified by preparative HPLC.

All of compound **5a-g** exist as rotamers due to restricted rotation around the amide bond this has been confirmed by VT-NMR and NOESY spectrum. In both the ^{1}H and ^{13}C NMR, only the major rotamer has been assigned. Integration for the NMR peaks includes both the major and minor rotamers to ensure accurate nuclei count. Where compounds exist as a mixture of diastereomers, it has been indicated if the assignment refers to both diastereomers or a single diastereomer. Diastereomers haven’t been separated and their ratio is approximate value.

(2R,4R)-1-(2-chloroacetyl)-N-(3-chlorophenyl)-4-phenylpyrrolidine-2-carboxamide (**5a, NU000846b**)

Isolated as yellow creme, 131.1 mg (95.3 %)

^{1}H NMR (400 MHz, DMSO-d_6)

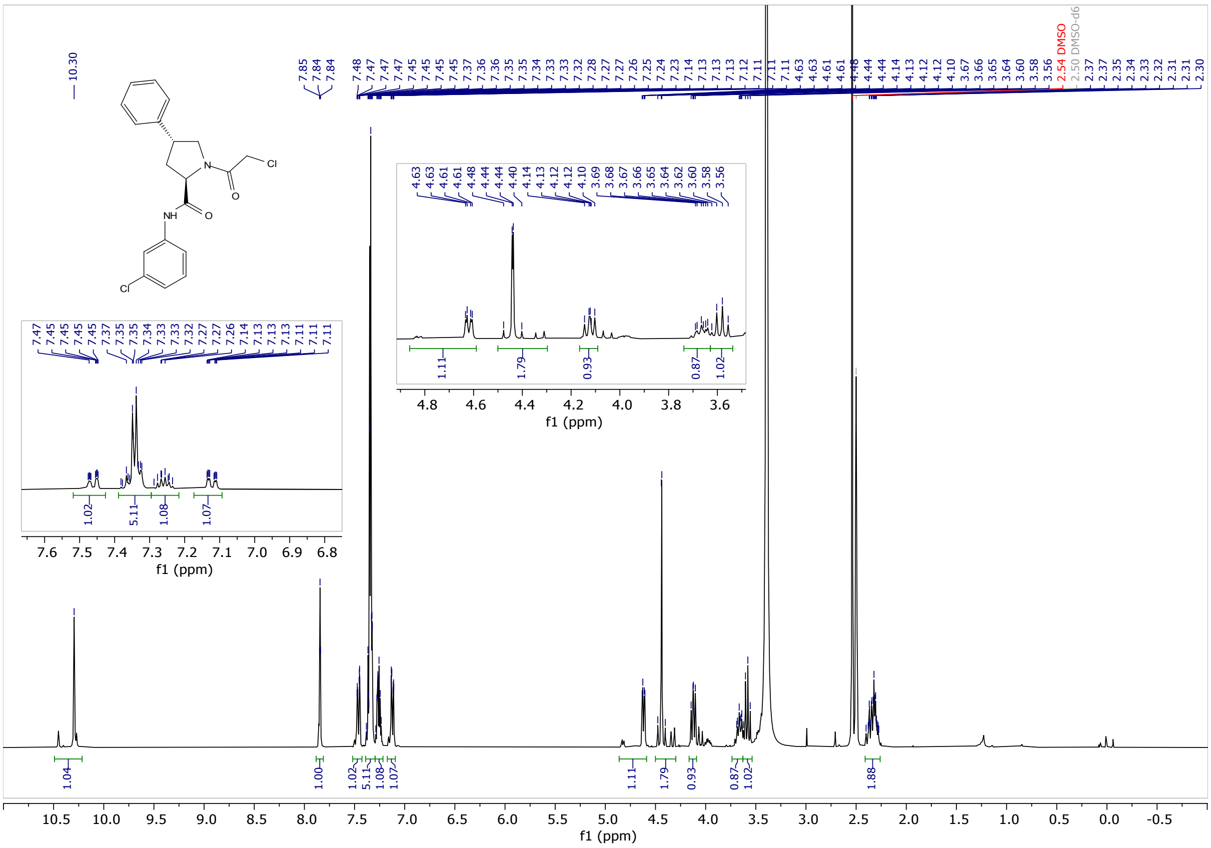

^{1}H NMR (400 MHz, DMSO-d_6) δ 10.30 (s, 1H), 7.84 (t, *J* = 2.1 Hz, 1H), 7.46 (ddd, *J* = 8.2, 2.1, 1.0 Hz, 1H), 7.39 – 7.30 (m, 5H), 7.30 – 7.23 (m, 1H), 7.12 (ddd, *J* = 8.0, 2.1, 1.0 Hz, 1H), 4.62 (dd, *J* = 8.4, 2.6 Hz, 1H), 4.49 – 4.39 (m, 2H), 4.12 (dd, *J* = 9.4, 7.4 Hz, 1H), 3.72 – 3.62 (m, 1H), 3.58 (t, *J* = 9.4 Hz, 1H), 2.41 – 2.26 (m, 2H).

^{13}C NMR (101 MHz, DMSO-d$_6$)

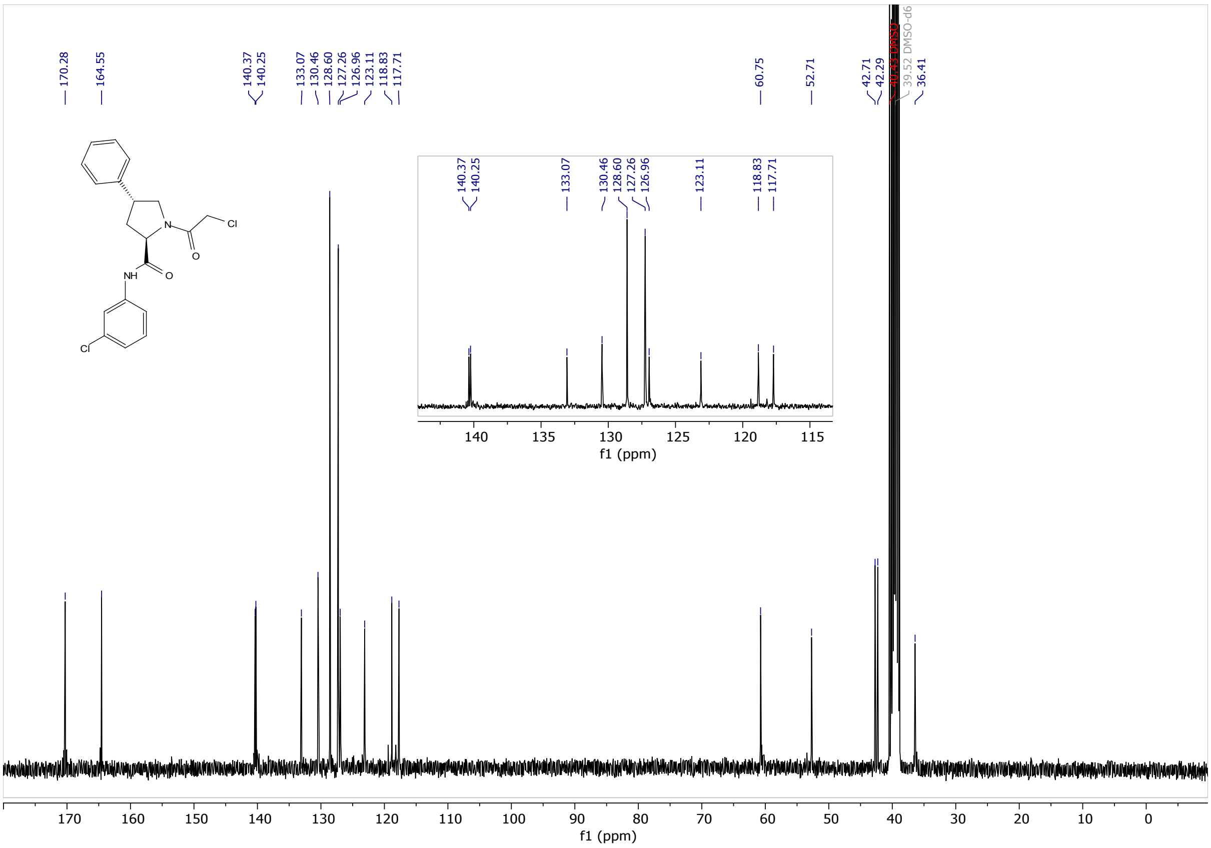

^{13}C NMR (101 MHz, DMSO-d$_6$) δ 170.3, 164.5, 140.4, 140.2, 133.1, 130.5, 128.6, 127.3, 123.1, 118.8, 117.7, 60.7, 52.7, 42.7, 42.3, 36.4.

LC-MS (ELSD, ESI)

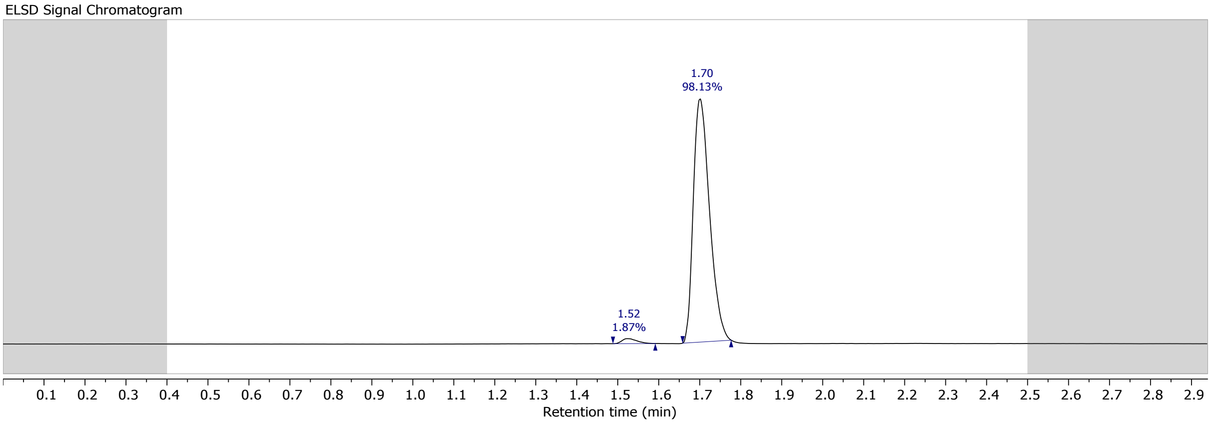

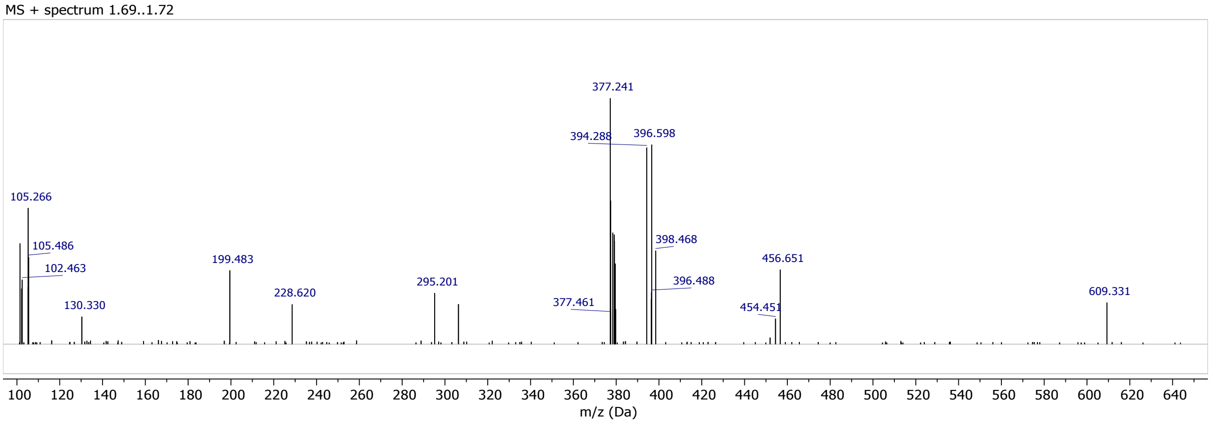

MS (ESI) for $[M + H]^+$ ($C_{19}H_{19}Cl_2N_2O_2^+$): calcd m/z 377.082; found m/z 377.241. LC–MS: R_t = 1.70, 98% purity in ELSD.

(2R,4R)-1-(2-chloroacetyl)-4-phenyl-N-(1,2,3,4-tetrahydronaphthalen-1-yl)pyrrolidine-2-carboxamide (**5b, NU074381b**)

Isolated as light brown crème, 70 mg (97 %), 70:30 diastereomeric mixture

^{1}H NMR (400 MHz, DMSO-d_6)

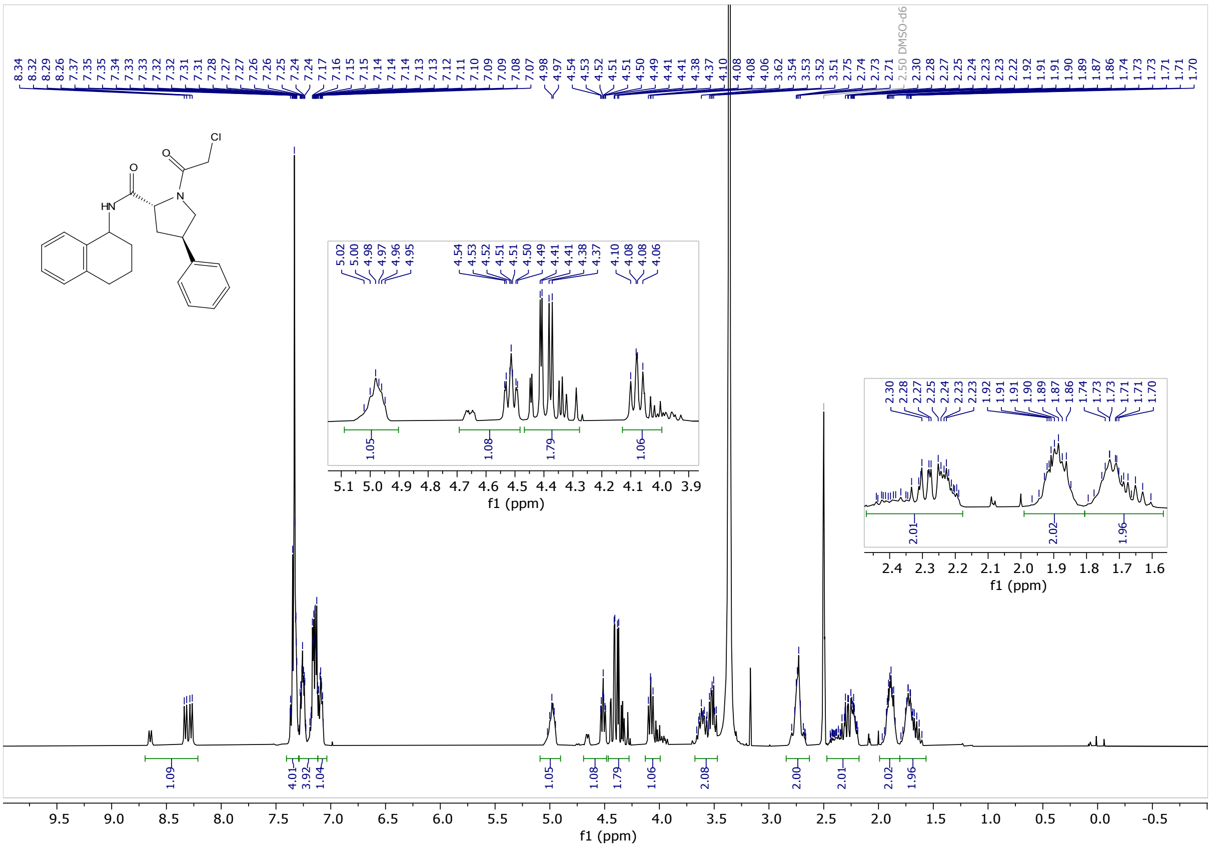

^{1}H NMR (400 MHz, DMSO-d_6) δ 8.33 (d, *J* = 8.9 Hz, 1H, single diastereomer), 8.28 (d, *J* = 8.7 Hz, 1H, single diastereomer), 7.39 – 7.29 (m, 4H), 7.25 (m, 1H), 7.20 – 7.12 (m, 3H), 7.11 – 7.06 (m, 1H), 5.07 – 4.91 (m, 1H), 4.51 (ddd, *J* = 8.7, 7.0, 2.2 Hz, 1H), 4.41 (d, *J* = 2.5 Hz, 2H, single diastereomer), 4.38 (d, *J* = 4.4 Hz, 2H, single diastereomer), 4.08 (dd, *J* = 9.3, 7.5 Hz, 1H), 3.68 – 3.57 (m, 1H), 3.52 (qd, *J* = 9.3, 5.8 Hz, 1H), 2.84 – 2.65 (m, 2H), 2.47 – 2.17 (m, 2H), 1.98 – 1.82 (m, 2H), 1.80 – 1.59 (m, 2H).

^{13}C NMR (101 MHz, DMSO-d_6)

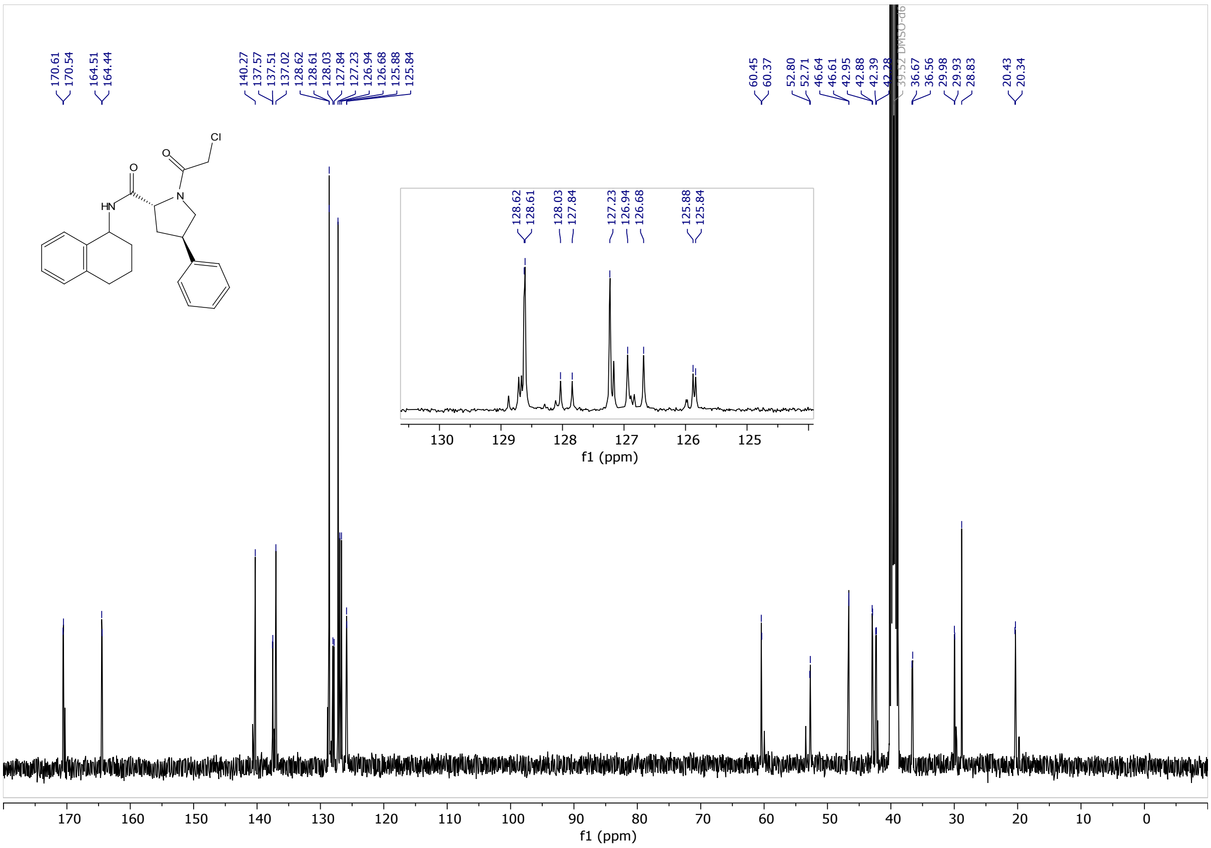

^{13}C NMR (101 MHz, DMSO-d_6) δ 170.6, 170.5, 164.5, 164.4, 140.3 (both diastereomers), 137.6, 137.5, 137.0 (both diastereomers), 128.62, 128.61, 128.0 (both diastereomers), 127.8 (both diastereomers), 127.2 (both diastereomers), 126.9 (both diastereomers), 126.7 (both diastereomers), 125.9, 125.8, 60.45, 60.37, 52.8, 52.7, 46.64, 46.61, 43.0, 42.9, 42.4, 42.3, 36.7, 36.6, 30.0, 29.9, 28.8 (both diastereomers), 20.4, 20.3.

LC-MS (220/254 nm, ESI)

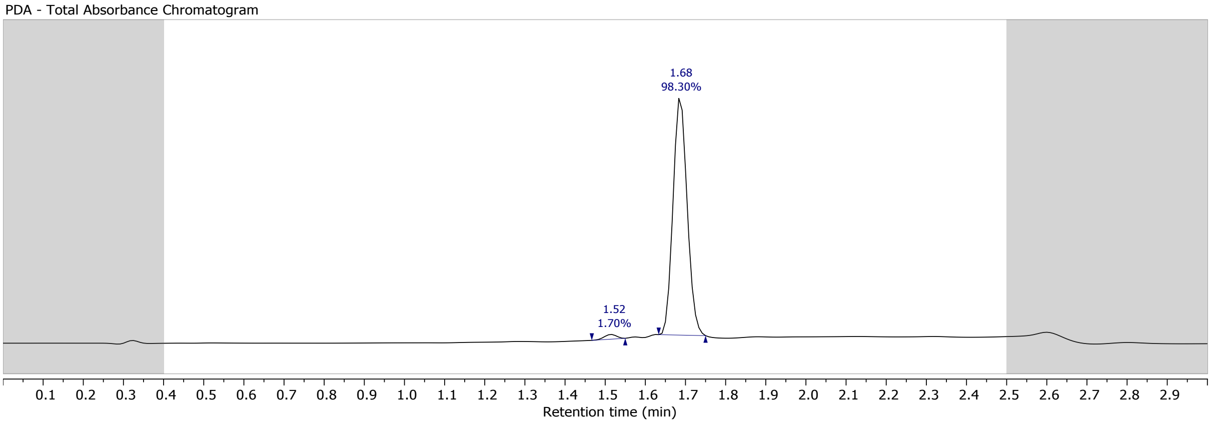

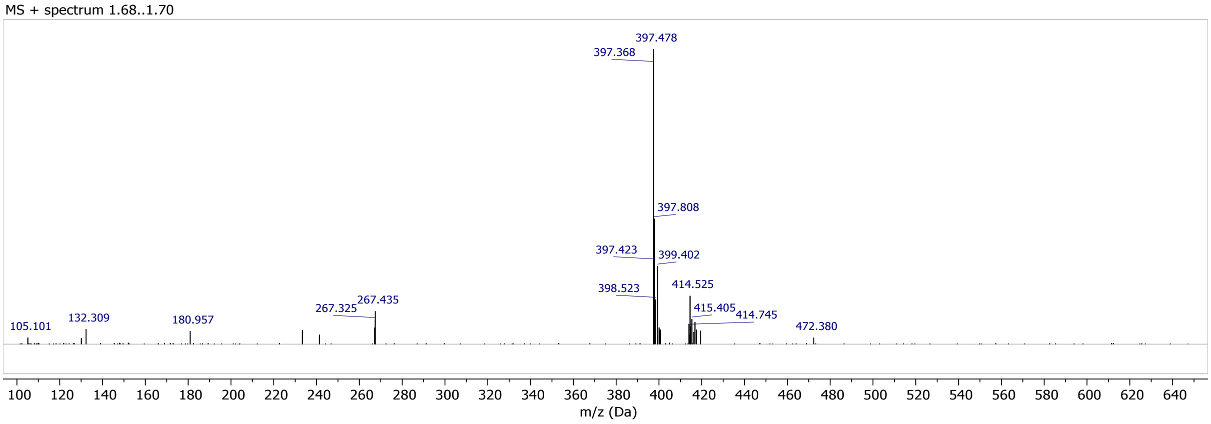

MS (ESI) for $[M + H]^+$ ($C_{23}H_{26}ClN_2O_2^+$): calcd m/z 397.168; found m/z 397.478. LC–MS: R_t = 1.68, 98% purity at 220/254 nm.

(2R,4R)-N-((R)-1-(1H-benzo[d]imidazol-2-yl)-3-methylbutyl)-1-(2-chloroacetyl)-4-phenylpyrrolidine-2-carboxamide (**5c, NU074391a**)

Isolated as colorless crème, 60.5mg (74 %)

^{1}H NMR (400 MHz, DMSO-d_6)

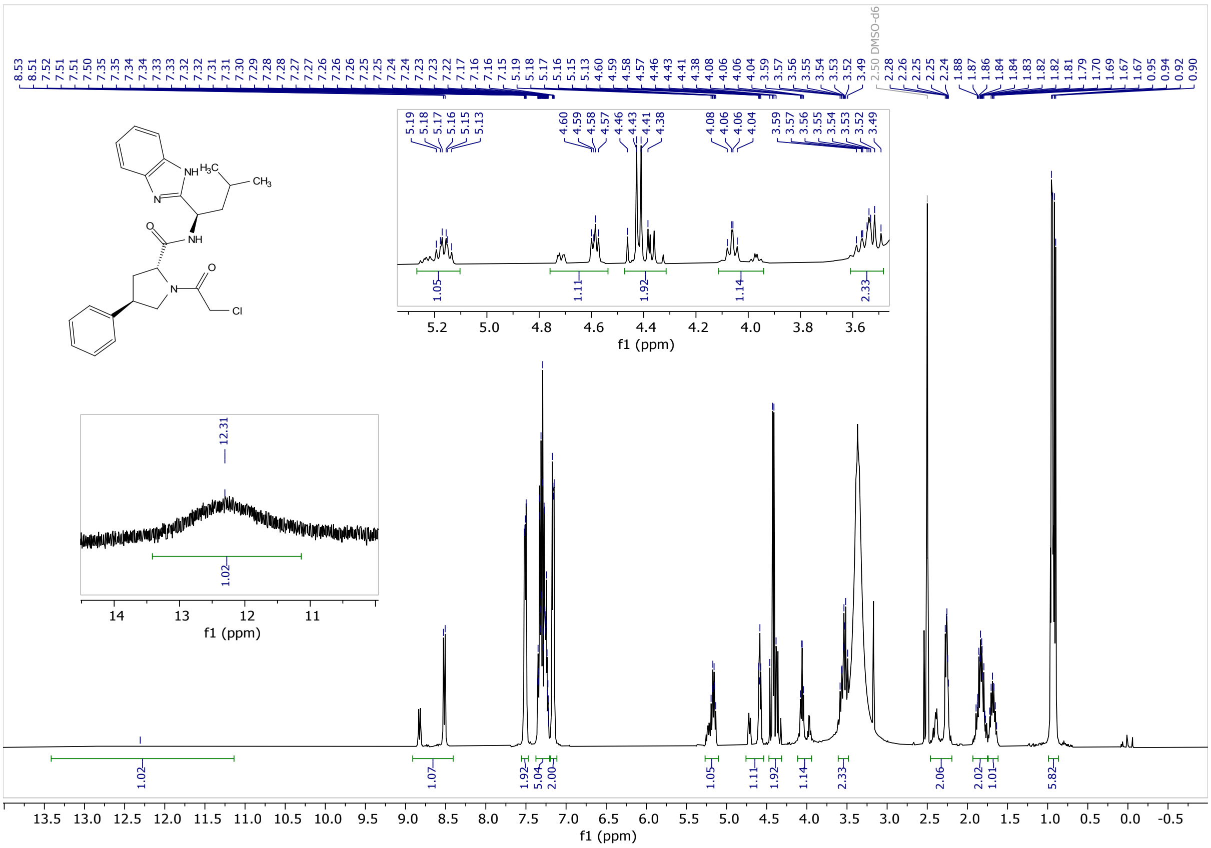

^{1}H NMR (400 MHz, DMSO-d_6) δ 12.31 (br s, 1H), 8.52 (d, *J* = 8.2 Hz, 1H), 7.51 (dd, *J* = 6.0, 3.3 Hz, 2H), 7.37 – 7.21 (m, 5H), 7.16 (dd, *J* = 6.0, 3.2 Hz, 2H), 5.16 (td, *J* = 8.7, 6.2 Hz, 1H), 4.61 – 4.56 (m, 1H), 4.47 – 4.38 (m, 2H), 4.09 – 4.03 (m, 1H), 3.63 – 3.48 (m, 2H), 2.29 – 2.22 (m, 2H), 1.91 – 1.77 (m, 2H), 1.74 – 1.62 (m, 1H), 0.95 (d, *J* = 6.6 Hz, 3H), 0.91 (d, *J* = 6.5 Hz, 3H).

^{13}C NMR (101 MHz, DMSO-d_6)

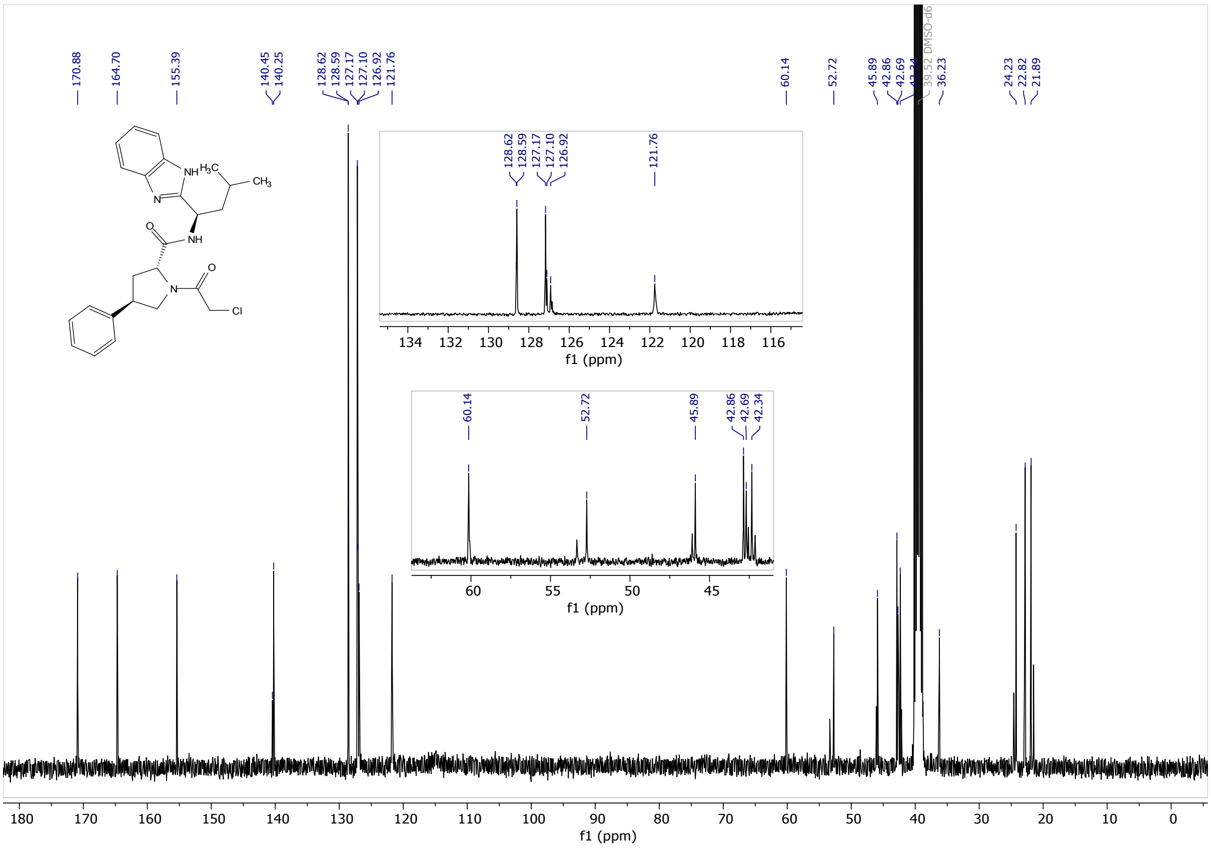

^{13}C NMR (101 MHz, DMSO-d_6) δ 170.9, 164.7, 155.4, 140.5, 140.2, 128.6, 128.6, 127.2, 127.1, 126.9, 121.8, 60.1, 52.7, 45.9, 42.9, 42.7, 42.3, 36.2, 24.2, 22.8, 21.9.

LC-MS (254 nm, ESI)

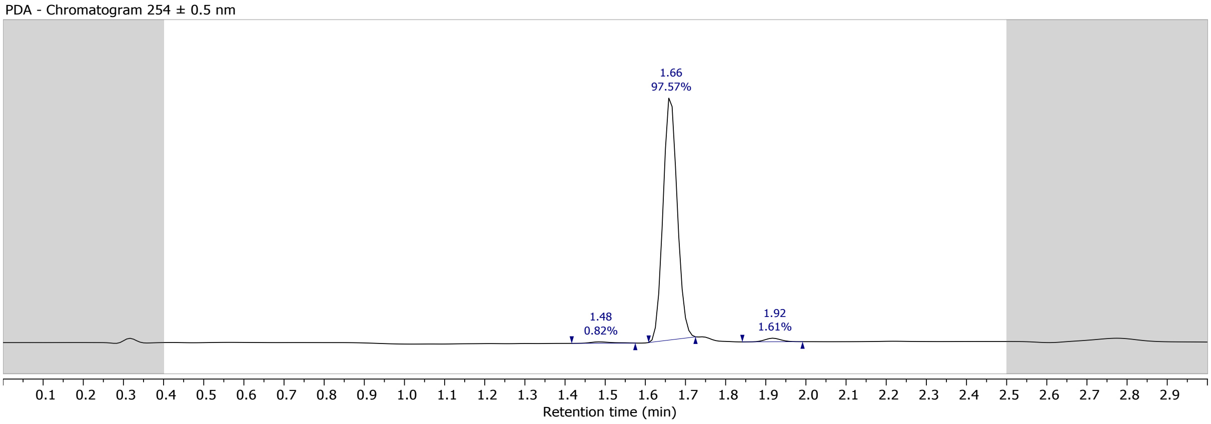

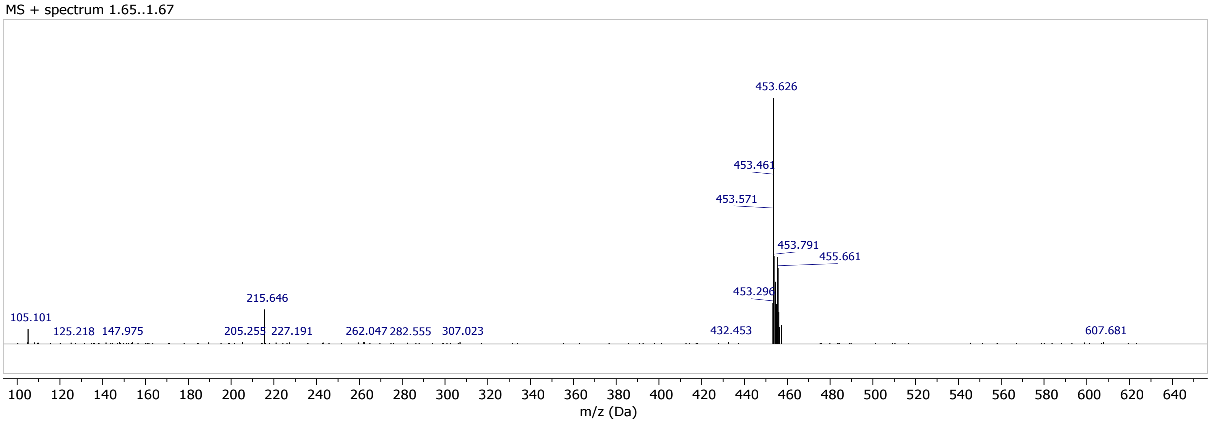

MS (ESI) for $[M + H]^+$ ($C_{25}H_{30}ClN_4O_2^+$): calcd m/z 453.205; found m/z 453.626. LC–MS: R_t = 1.66, 98% purity at 254 nm.

(2R,4R)-1-(2-chloroacetyl)-N-(1-methyl-2-oxopiperidin-3-yl)-4-phenylpyrrolidine-2-carboxamide (**5d, NU074379b**)

Isolated as white crème, 53.9 mg (78.2 %)

^{1}H NMR (400 MHz, DMSO-d_6)

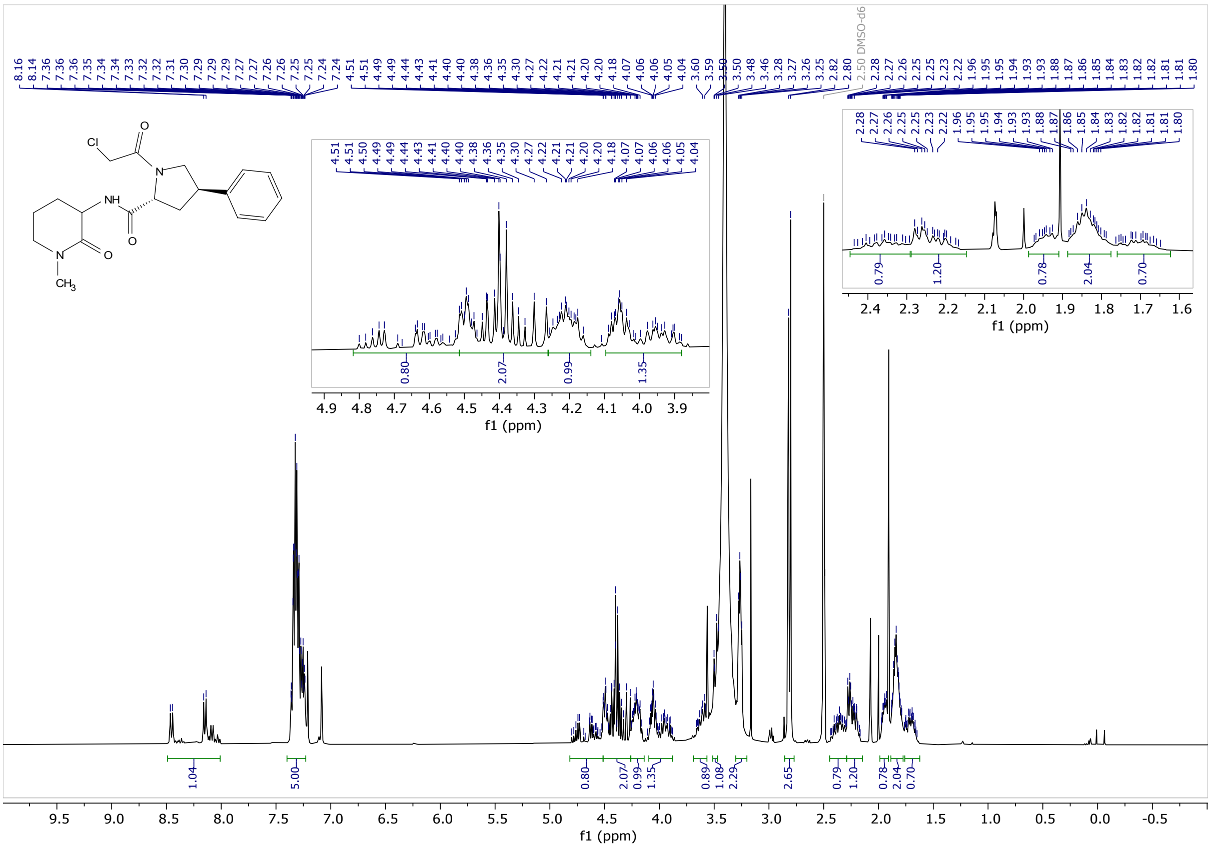

^{1}H NMR (400 MHz, DMSO-d_6) δ 8.45 (d, J = 8.5 Hz, 1H, single diastereomer), 8.15 (d, J = 8.5 Hz, 1H, single diastereomer), 7.39 – 7.22 (m, 5H), 4.82 – 4.53 (m, 1H), 4.53 – 4.26 (m, 2H), 4.26 – 4.15 (m, 1H), 4.10 – 4.00 (m, 1H, single diastereomer), 4.00 – 3.87 (m, 1H, single diastereomer), 3.68 – 3.57 (m, 1H), 3.51 – 3.47 (m, 1H), 3.29 – 3.23 (m, 2H), 2.82 (s, 3H, single diastereomer), 2.80 (s, 3H, single diastereomer), 2.44 – 2.30 (m, 2H, single diastereomer), 2.29 – 2.15 (m, 2H, single diastereomer), 1.98 – 1.91 (m, 1H), 1.89 – 1.78 (m, 2H), 1.76 – 1.63 (m, 1H).

^{13}C NMR (101 MHz, DMSO-d_6)

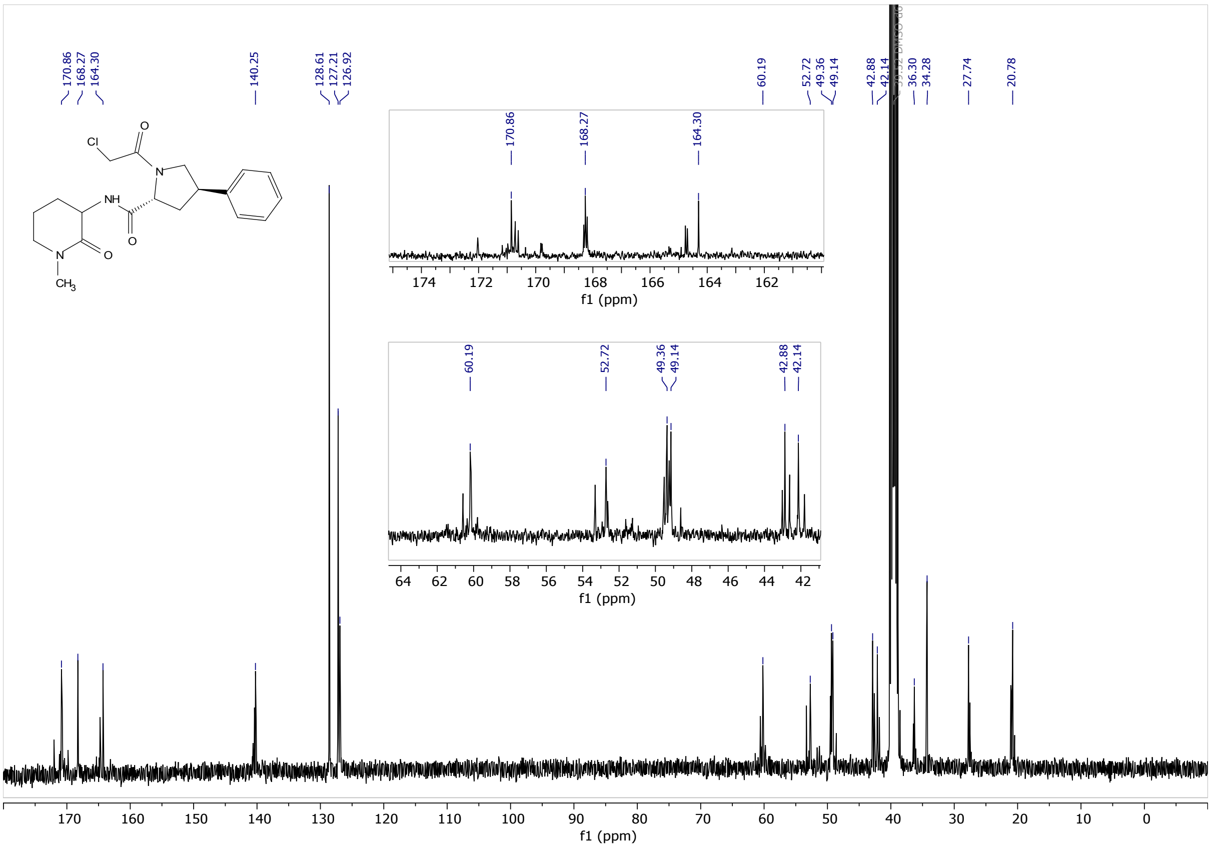

^{13}C NMR (101 MHz, DMSO-d_6) δ 170.9, 168.3, 164.3, 140.2, 128.6, 127.2, 126.9, 60.2, 52.7, 49.4, 49.1, 42.9, 42.1, 36.3, 34.3, 27.7, 20.8. Only the major diastereomer is assigned.

LC-MS (220/254 nm, ESI)

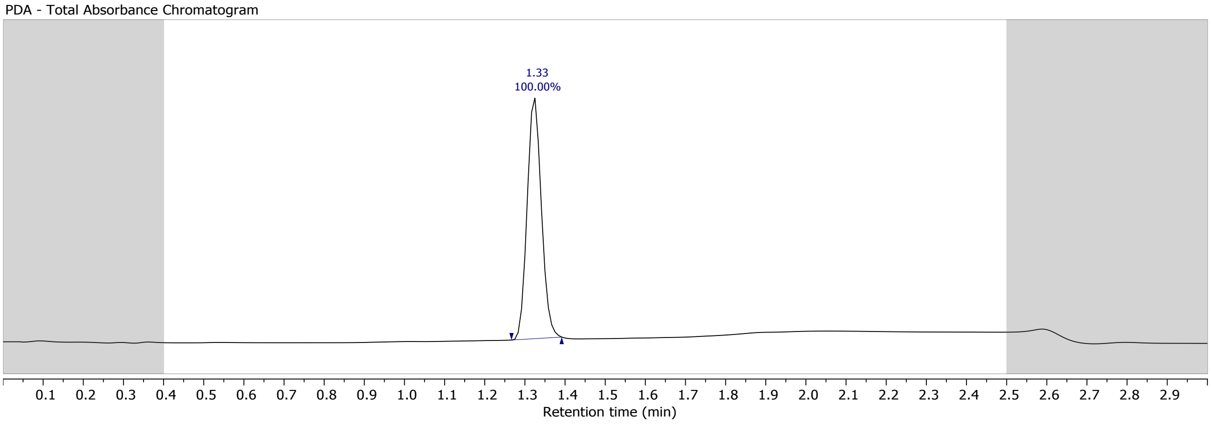

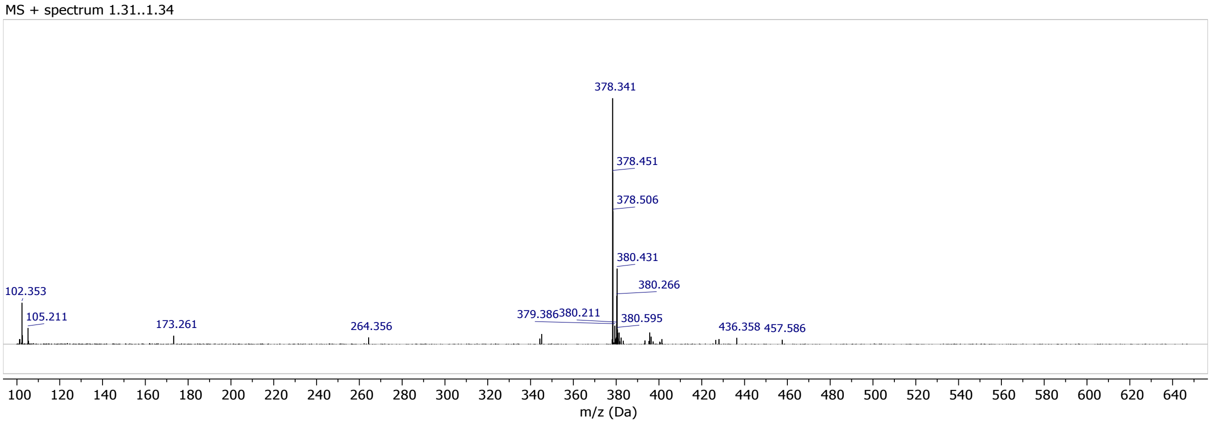

MS (ESI) for $[M + H]^+$ ($C_{19}H_{25}ClN_3O_3^+$): calcd m/z 378.158; found m/z 378.341. LC–MS: R_t = 1.33, 100% purity at 220/254 nm.

(2R,4R)-1-(2-chloroacetyl)-N-(2-oxoindolin-3-yl)-4-phenylpyrrolidine-2-carboxamide (**5e, NU074382b**)

Isolated as white crème, 61.3 mg (84.7 %)

^{1}H NMR (400 MHz, DMSO-d_6)

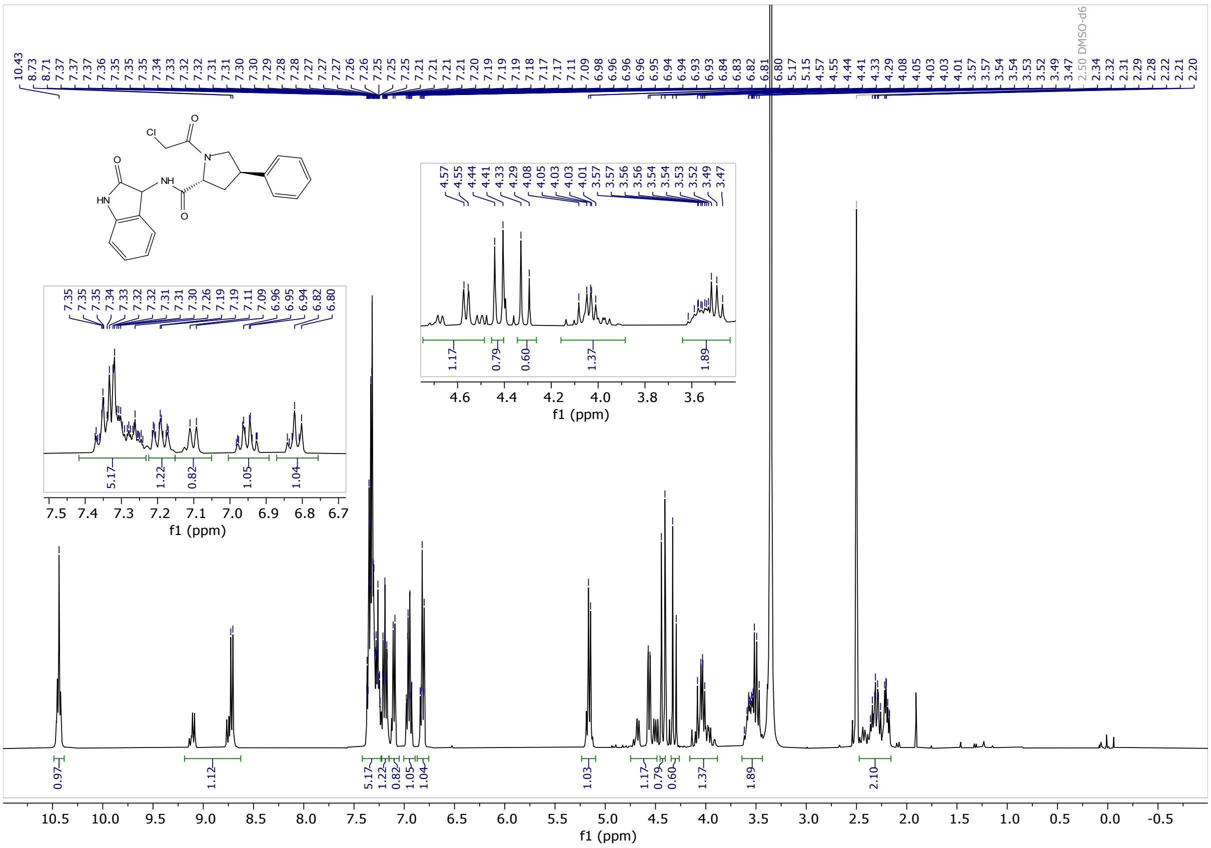

^{1}H NMR (400 MHz, DMSO-d_6) δ 10.43 (s, 1H), 8.72 (d, *J* = 8.2 Hz, 1H), 7.39 – 7.24 (m, 5H), 7.22 – 7.16 (m, 1H), 7.10 (d, *J* = 7.0 Hz, 1H), 7.00 – 6.91 (m, 1H), 6.82 (t, *J* = 7.8 Hz, 1H), 5.16 (d, *J* = 8.2 Hz, 1H), 4.56 (d, *J* = 7.1 Hz, 1H), 4.42 (d, *J* = 14.1 Hz, 1H), 4.31 (d, *J* = 14.1 Hz, 1H), 4.09 – 4.00 (m, 1H), 3.62 – 3.45 (m, 2H), 2.38 – 2.15 (m, 2H). Only the major diastereomer is assigned.

^{13}C NMR (101 MHz, DMSO-d$_6$)

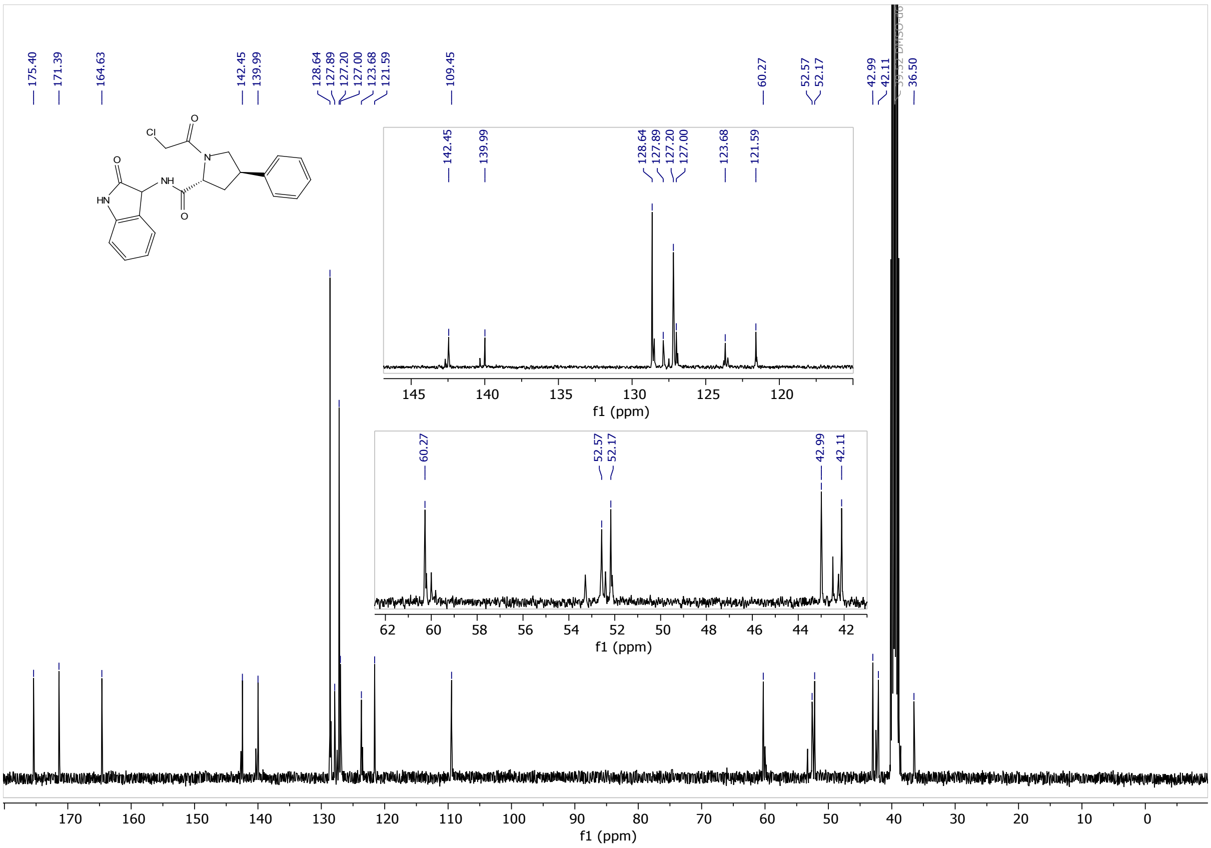

^{13}C NMR (101 MHz, DMSO-d$_6$) δ 175.4, 171.4, 164.6, 142.5, 140.0, 128.6, 127.9, 127.2, 127.0, 123.7, 121.6, 109.5, 60.3, 52.6, 52.2, 43.0, 42.1, 36.5. Only the major diastereomer is assigned.

LC-MS (254 nm, ESI)

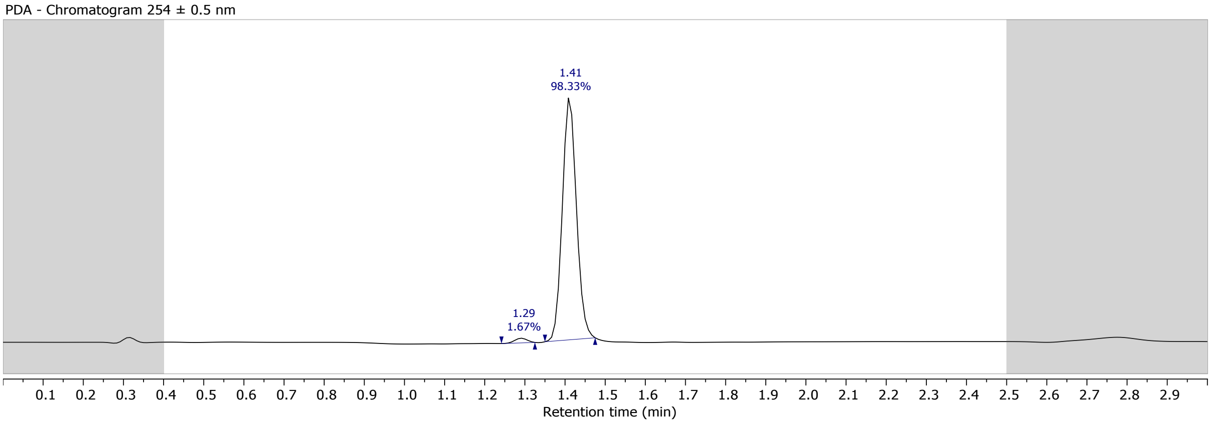

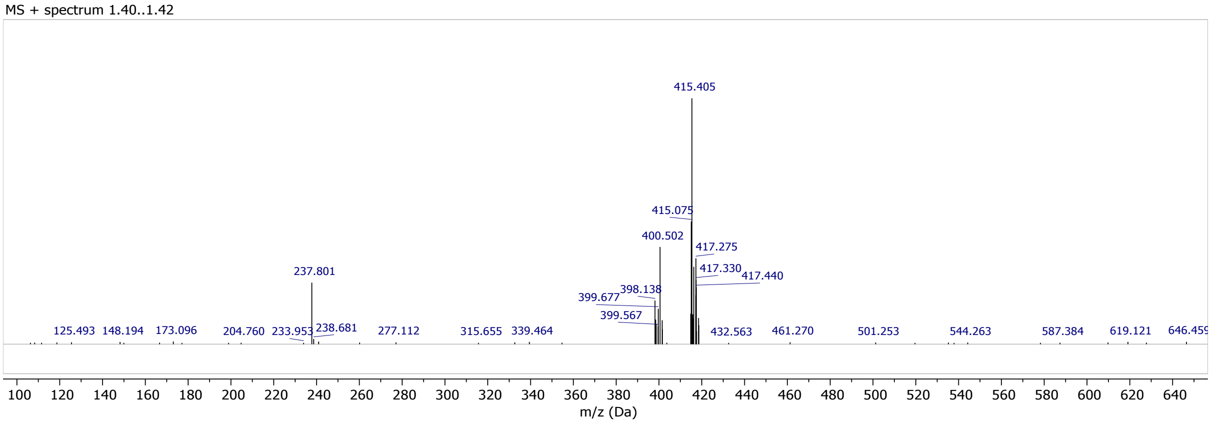

MS (ESI) for $[M + NH_4]^+$ ($C_{21}H_{24}ClN_4O_3^+$): calcd m/z 415.153; found m/z 415.405. LC–MS: R_t = 1.41, 98% purity at 254 nm.

(2R,4R)-1-(2-chloroacetyl)-N-(1-(3-methoxyphenyl)-5-oxopyrrolidin-3-yl)-4-phenylpyrrolidine-2-carboxamide (**5f, NU074390a**)

Isolated as white crème, 77 mg (93.7 %)

^{1}H NMR (400 MHz, DMSO-d_6)

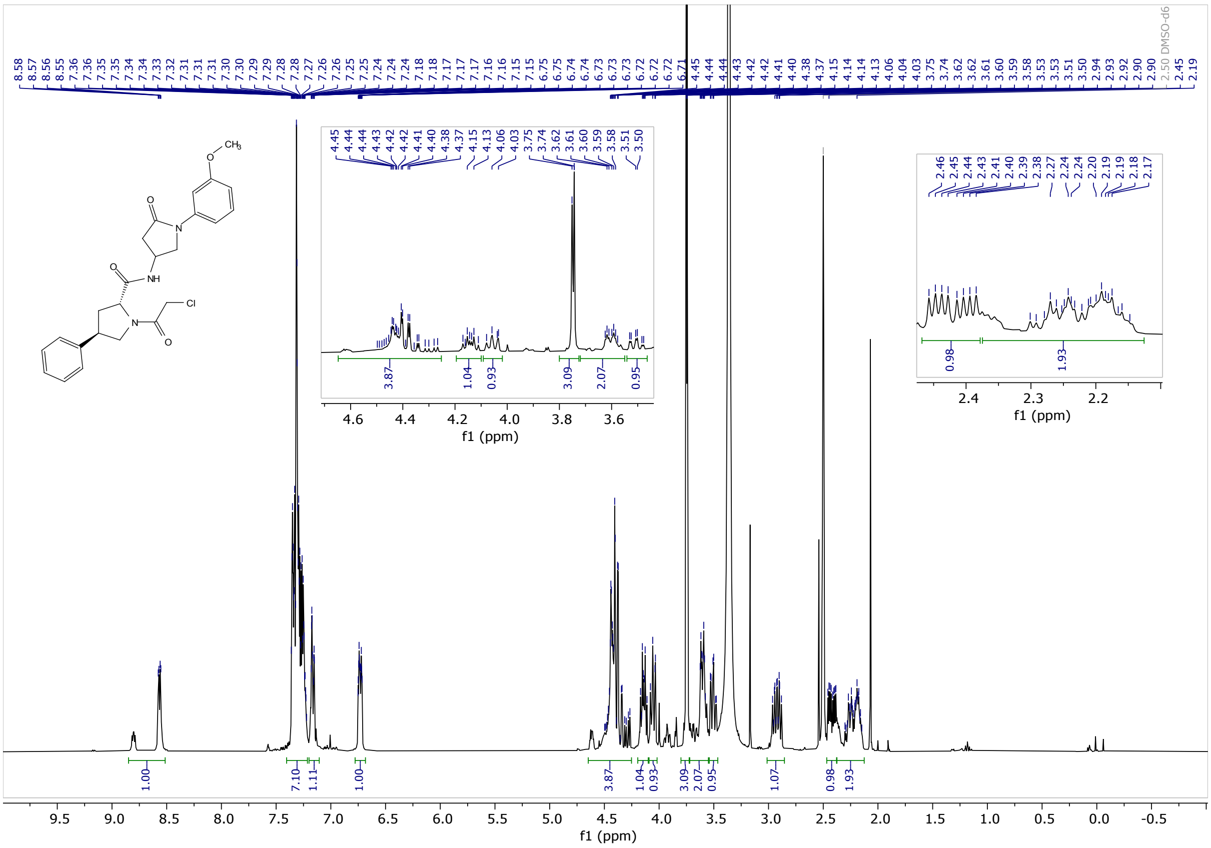

^{1}H NMR (400 MHz, DMSO-d_6) δ 8.57 (d, *J* = 7.0 Hz, 1H, single diastereomer), 8.56 (d, *J* = 7.0 Hz, 1H, single diastereomer), 7.38 – 7.22 (m, 7H), 7.18 – 7.14 (m, 1H), 6.77 – 6.69 (m, 1H), 4.54 – 4.24 (m, 4H), 4.14 (dt, *J* = 10.3, 6.8 Hz, 1H), 4.09 – 4.02 (m, 1H), 3.75 (s, 3H, single diastereomer), 3.74 (s, 3H, single diastereomer), 3.65 – 3.57 (m, 2H), 3.50 (td, *J* = 9.5, 2.5 Hz, 1H), 2.99 – 2.87 (m, 1H), 2.42 (ddd, *J* = 17.3, 7.9, 3.9 Hz, 1H), 2.34 – 2.13 (m, 2H).

^{13}C NMR (101 MHz, DMSO-d_6)

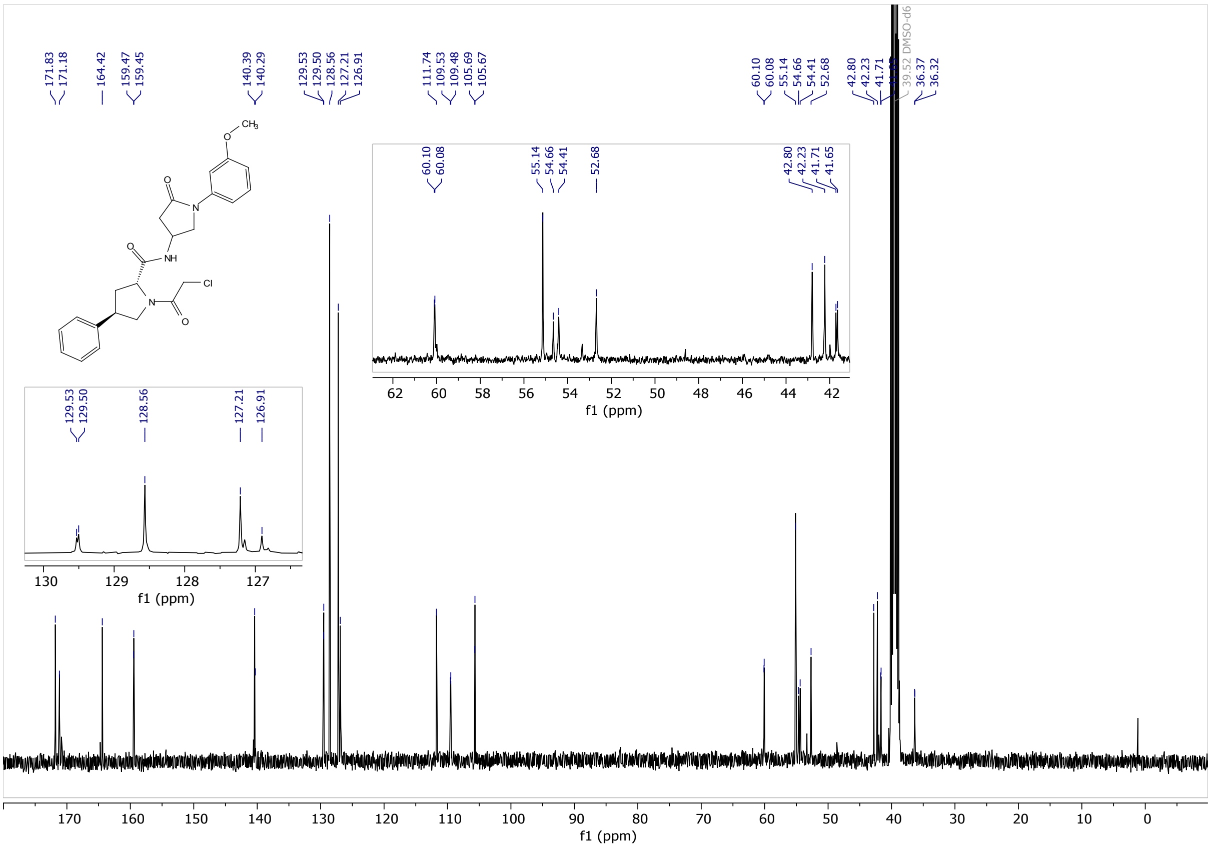

^{13}C NMR (101 MHz, DMSO-d_6) δ 171.8, 171.2, 164.4, 159.5 (single diastereomer), 159.4 (single diastereomer), 140.4, 140.3, 129.53 (single diastereomer), 129.50 (single diastereomer), 128.6, 127.2 , 126.9, 111.7, 109.53 (single diastereomer), 109.48 (single diastereomer), 105.69 (single diastereomer), 105.67 (single diastereomer), 60.10 (single diastereomer), 60.08 (single diastereomer), 55.1, 54.7, 54.4, 52.7, 42.8, 42.2, 41.7 (single diastereomer), 41.6 (single diastereomer), 36.4 (single diastereomer), 36.3 (single diastereomer).

LC-MS (220/254 nm, ESI)

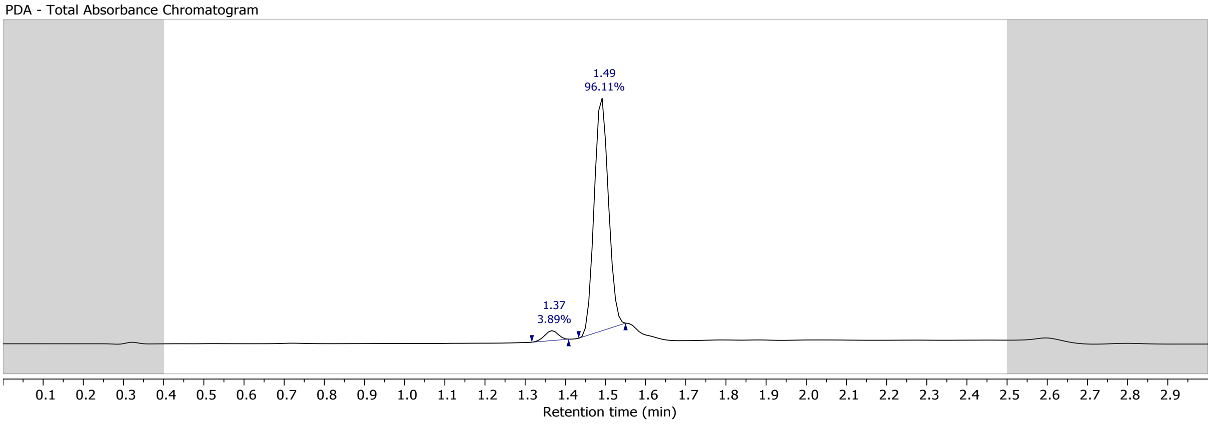

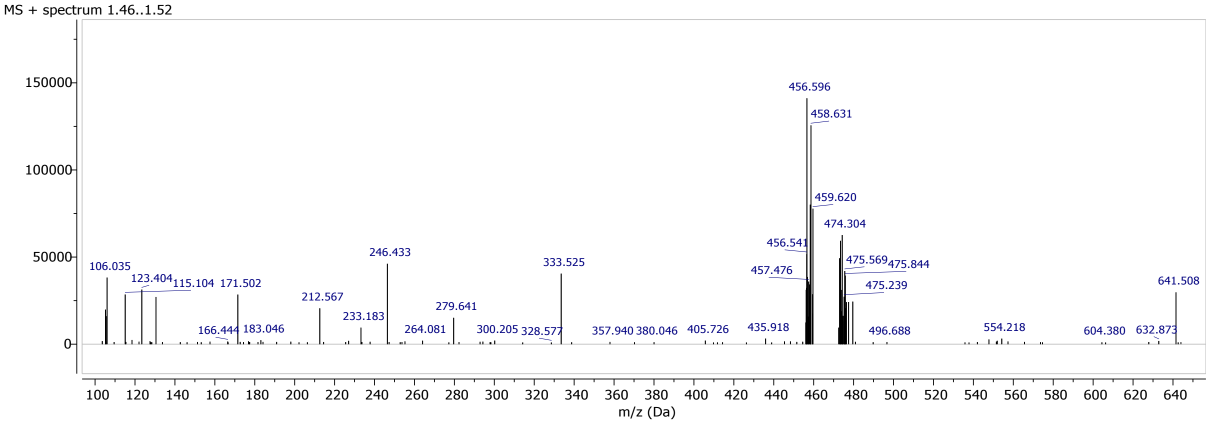

MS (ESI) for [M + H]$^+$ ($C_{21}H_{24}ClN_4O_3^+$): calcd m/z 456.168; found m/z 456.596. LC–MS: R_t = 1.49, 96% purity at 220/254 nm.

(2R,4R)-1-(2-chloroacetyl)-N-(4-(cyanomethyl)phenyl)-4-phenylpyrrolidine-2-carboxamide (**5g, NU074392a**)

Isolated as white crème, 69 mg (99.2 %)

^{1}H NMR (400 MHz, DMSO-d_6)

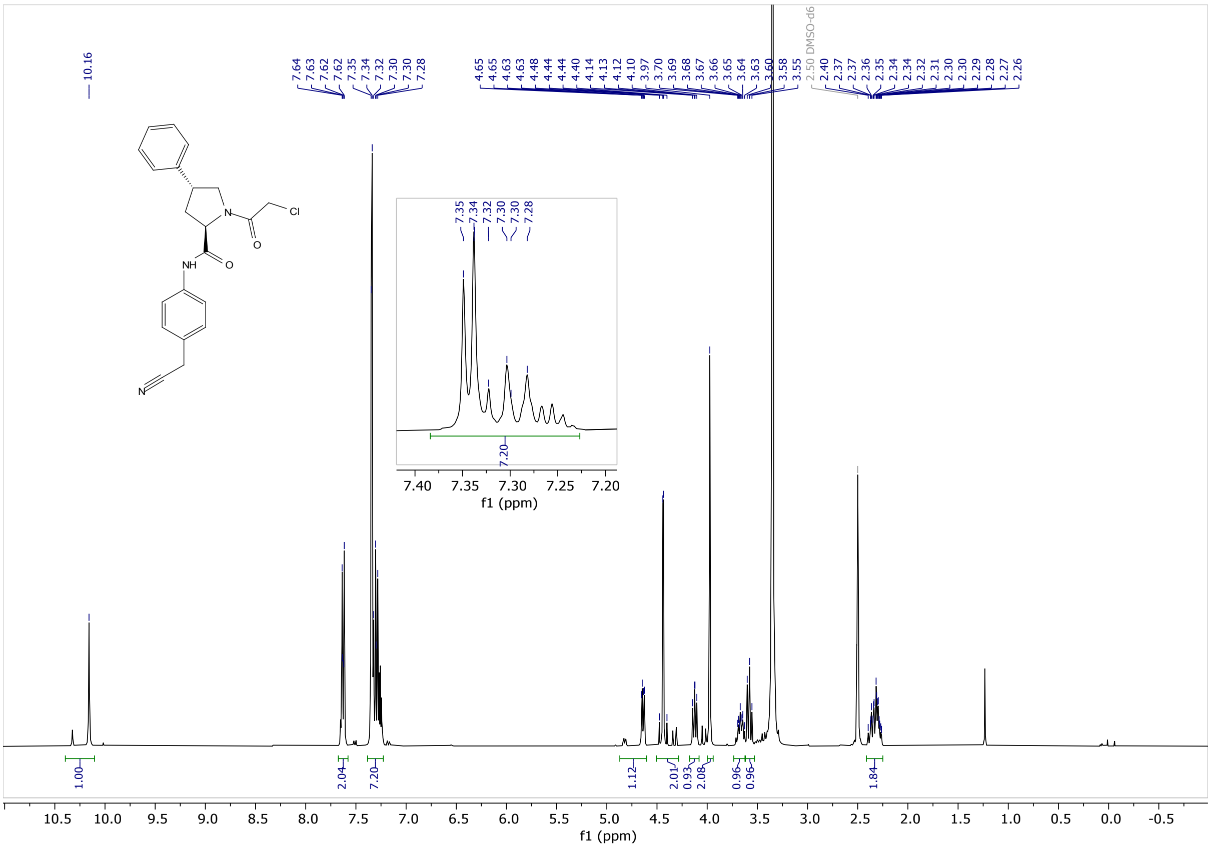

^{1}H NMR (400 MHz, DMSO-d_6) δ 10.16 (s, 1H), 7.65 – 7.60 (m, 2H), 7.38 – 7.27 (m, 7H), 4.64 (dd, *J* = 8.4, 2.5 Hz, 1H), 4.49 – 4.39 (m, 2H), 4.12 (dd, *J* = 9.3, 7.4 Hz, 1H), 3.97 (s, 2H), 3.72 – 3.62 (m, 1H), 3.58 (t, *J* = 9.5 Hz, 1H), 2.41 – 2.25 (m, 2H).

^{13}C NMR (101 MHz, DMSO-d_6)

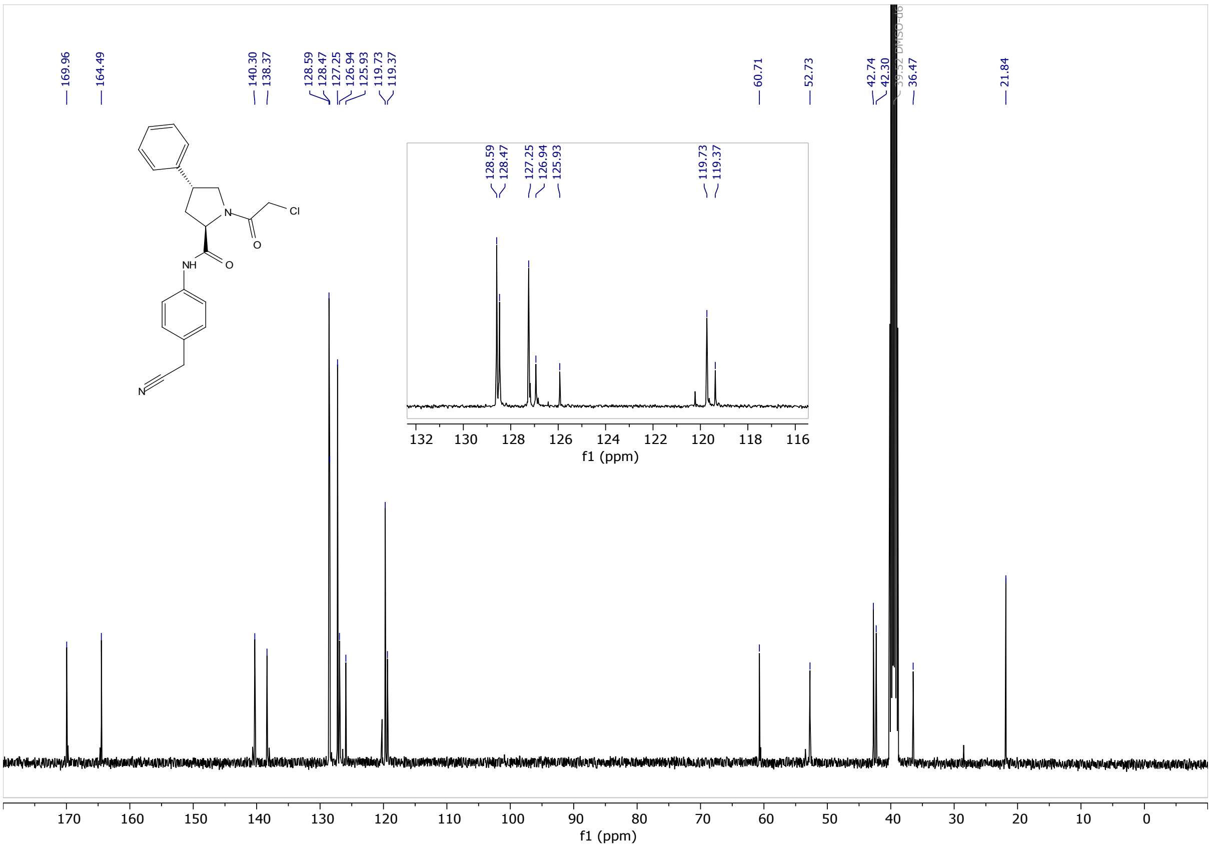

^{13}C NMR (101 MHz, DMSO-d_6) δ 170.0, 164.5, 140.3, 138.4, 128.6, 128.5, 127.2, 126.9, 125.9, 119.7, 119.4, 60.7, 52.7, 42.7, 42.3, 36.5, 21.8.

LC-MS (254 nm, ESI)

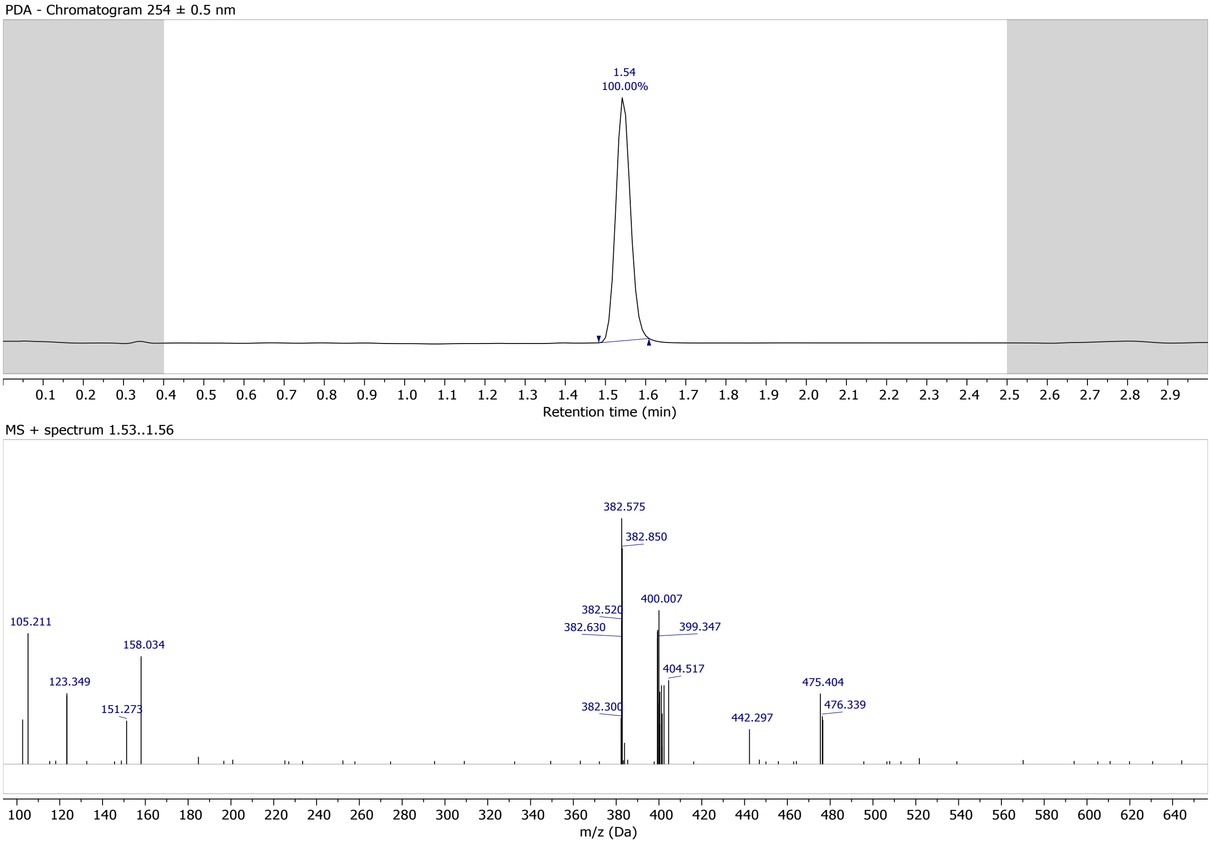

MS (ESI) for $[M + H]^+$ ($C_{21}H_{21}ClN_3O_2^+$): calcd m/z 382.132; found m/z 382.575. LC–MS: R_t = 1.54, 100% purity at 254 nm.

Table S4: Compound IDs and SMILES

ACS ID	Compound ID	SMILES
**1**	PK006912b	Cc1ccc(cc1)S(C=CC#N)(=O)=O
**2a**	KD036433a	CC(C)c1c(-c2ccc(OCCNC(=O)C=CC(=O)c3ccccc3)cc2)[nH]c2c(C#N)cnn2c1=O
**2b**	KD036434a	CC(C)c1c(-c2cnn(C3CCN(C(=O)C=CC(=O)c4ccccc4)CC3)c2)[nH]c2c(C#N)cnn2c1=O
**2c**	KD036432a	CC(C)c1c(-c2cnn(CCNC(=O)C=CC(=O)c3ccccc3)c2)[nH]c2c(C#N)cnn2c1=O
**2d**	KD036425a	[H]N1C=Nc2c(ccnc2n2cc(CCN3CCc4ccc(cc4C3)N([H])C(C[Cl])=O)cn2)C1=O
**3a**	KD036424a	[H]N1C=Nc2c(ccnc2n2cc(CCN(Cc3cccc(c3)N([H])C(C[Cl])=O)C3CCCC3)cn2)C1=O
**3b**	KD033833a	[H]N1C=Nc2c(ccnc2n2cc(CCN(Cc3cccc(c3)N([H])C(C=C)=O)C3CCCC3)cn2)C1=O
**4a**	KD033835a	[H]N1C=Nc2c(ccnc2n2cc(CCN3CCN(CC3)C(C#C)=O)cn2)C1=O
**5**	NU000846a	O=C(Nc1cccc(Cl)c1)[C@H]1C[C@H](c2ccccc2)CN1C(=O)CCl
**5a**	NU000846b	O=C(Nc1cccc(Cl)c1)[C@H]1C[C@H](c2ccccc2)CN1C(=O)CCl
**5b**	NU074381b	O=C(NC1CCCc2ccccc21)[C@H]1C[C@H](c2ccccc2)CN1C(=O)CCl
**5c**	NU074391a	CC(C)C[C@@H](NC(=O)[C@H]1C[C@H](c2ccccc2)CN1C(=O)CCl)c1nc2ccccc2[nH]1
**5d**	NU074379b	CN1CCCC(NC(=O)[C@H]2C[C@H](c3ccccc3)CN2C(=O)CCl)C1=O
**5e**	NU074382b	O=C1Nc2ccccc2C1NC(=O)[C@H]1C[C@H](c2ccccc2)CN1C(=O)CCl
**5f**	NU074390a	COc1cccc(N2CC(NC(=O)[C@H]3C[C@H](c4ccccc4)CN3C(=O)CCl)CC2=O)c1
**5g**	NU074392a	N#CCc1ccc(NC(=O)[C@H]2C[C@H](c3ccccc3)CN2C(=O)CCl)cc1

Table S5: Physicochemical and molecular properties

**Compound**	**5a**	**5b**	**5c**
molecular weight	376.07	396.16	452.2
LogD	3.95	3.74	4.64
**ADME profile**[#]			
aq. solubility *(µg/mL)/(µM)*	23.9/63.4	45.7/115	1.69/3.73
MLM, Cl_i *(µL/min/mg protein)* /	332 / 4*	778 / 2*	209 / 7*
MDR1-MDCK II, AB/BA P_{app}	0.8 / <26.4	3.8 / 34.5	12.6 / 38.8
MDR1-MDCK II, efflux ratio (ER)	<3.62[&]	0.3[&]	2.1[&]

[#]Studies were performed by WuXi AppTec (HongKong) Limited

[*]Possibly non-NADPH dependent metabolism

[&]The insufficient recovery (<50.0) might be caused by non-specific binding, cellular metabolism, cellular retention or other issues.

Crystallography

Table S6: Data collection and refinement statistics.

	S100A4-5a	S100A4-5b
**Wavelength**	0.9794 A	0.9763 A
**Resolution range**	47.43 - 2.61 (2.703 - 2.61)	53.82 - 1.91 (1.978 - 1.91)
**Space group**	P 64 2 2	P 2 21 21
**Unit cell**	109.54 109.54 89.87 90 90 120	64.913 70.404 107.64 90 90 90
**Total reflections**	20273 (1948)	
**Unique reflections**	10137 (974)	38994 (3820)
**Multiplicity**	2.0 (2.0)	
**Completeness (%)**	99.90 (100.00)	96.85 (69.37)
**Wilson B-factor**	87.58	49.23
**Reflections used in refinement**	10135 (974)	37770 (2650)
**Reflections used for R-free**	487 (43)	1791 (136)
**R-work**	0.2702 (0.3858)	0.2365 (0.4764)
**R-free**	0.3259 (0.4573)	0.2581 (0.5266)
**Number of non-hydrogen atoms**	1484	3077
**macromolecules**	1429	3001
**ligands**	52	62
**solvent**	3	14
**Protein residues**	195	402
**RMS(bonds)**	0.012	0.006
**RMS(angles)**	1.54	0.86
**Ramachandran favored (%)**	85.26	95.64
**Ramachandran allowed (%)**	10.53	4.1
**Ramachandran outliers (%)**	4.21	0.26
**Rotamer outliers (%)**	6.99	2.46
**Clashscore**	37.52	6.5
**Average B-factor**	99	55.14
**macromolecules**	98.45	54.84
**ligands**	114.89	70.24
**solvent**	82.82	51.44
